# A dual regulatory circuit consisting of *S*-adenosylmethionine decarboxylase protein and its reaction product controls expression of the paralogous activator prozyme in *Trypanosoma brucei*

**DOI:** 10.1371/journal.ppat.1007404

**Published:** 2018-10-26

**Authors:** Manish M. Patel, Oleg A. Volkov, Christopher Leija, Andrew Lemoff, Margaret A. Phillips

**Affiliations:** Department of Biochemistry, University of Texas Southwestern Medical Center at Dallas, Harry Hines Blvd, Dallas, TX, United States of America; University of California, Los Angeles, UNITED STATES

## Abstract

Polyamines are essential for cell growth of eukaryotes including the etiologic agent of human African trypanosomiasis (HAT), *Trypanosoma brucei*. In trypanosomatids, a key enzyme in the polyamine biosynthetic pathway, *S*-adenosylmethionine decarboxylase (*Tb*AdoMetDC) heterodimerizes with a unique catalytically-dead paralog called prozyme to form the active enzyme complex. In higher eukaryotes, polyamine metabolism is subject to tight feedback regulation by spermidine-dependent mechanisms that are absent in trypanosomatids. Instead, in *T*. *brucei* an alternative regulatory strategy based on *Tb*AdoMetDC prozyme has evolved. We previously demonstrated that prozyme protein levels increase in response to loss of *Tb*AdoMetDC activity. Herein, we show that prozyme levels are under translational control by monitoring incorporation of deuterated leucine into nascent prozyme protein. We furthermore identify pathway factors that regulate *prozyme* mRNA translation. We find evidence for a regulatory feedback mechanism in which *Tb*AdoMetDC protein and decarboxylated AdoMet (dcAdoMet) act as suppressors of *prozyme* translation. In *Tb*AdoMetDC null cells expressing the human AdoMetDC enzyme, prozyme levels are constitutively upregulated. Wild-type prozyme levels are restored by complementation with either *Tb*AdoMetDC or an active site mutant, suggesting that *Tb*AdoMetDC possesses an enzyme activity-independent function that inhibits prozyme translation. Depletion of dcAdoMet pools by three independent strategies: inhibition/knockdown of *Tb*AdoMetDC, knockdown of AdoMet synthase, or methionine starvation, each cause prozyme upregulation, providing independent evidence that dcAdoMet functions as a metabolic signal for regulation of the polyamine pathway in *T*. *brucei*. These findings highlight a potential regulatory paradigm employing enzymes and pseudoenzymes that may have broad implications in biology.

## Author summary

*Trypanosoma brucei* is a single-celled eukaryotic pathogen and the causative agent of human African trypanosomiasis (HAT). Polyamines are organic polycations that are essential for growth in *T*. *brucei* to facilitate protein translation and to maintain redox homeostasis. The pathway is the target of eflornithine, a current frontline therapy for treatment of HAT. Polyamine biosynthetic enzymes are regulated at multiple levels in mammals (e.g. transcription, translation and protein turnover), but in contrast, *T*. *brucei* lacks these mechanisms. Instead in *T*. *brucei* a central enzyme in polyamine metabolism called AdoMetDC must form a complex with a sister protein (termed a pseudoenzyme) to be active. Herein, we show that cellular levels of this sister protein we call prozyme are in turn feedback regulated by both AdoMetDC and by its reaction product in response to cell treatments that reduce pathway output. This regulatory paradigm highlights how pseudoenzymes can evolve to play an important role in metabolic pathway regulation and in organismal fitness.

## Introduction

**Fig 1:**
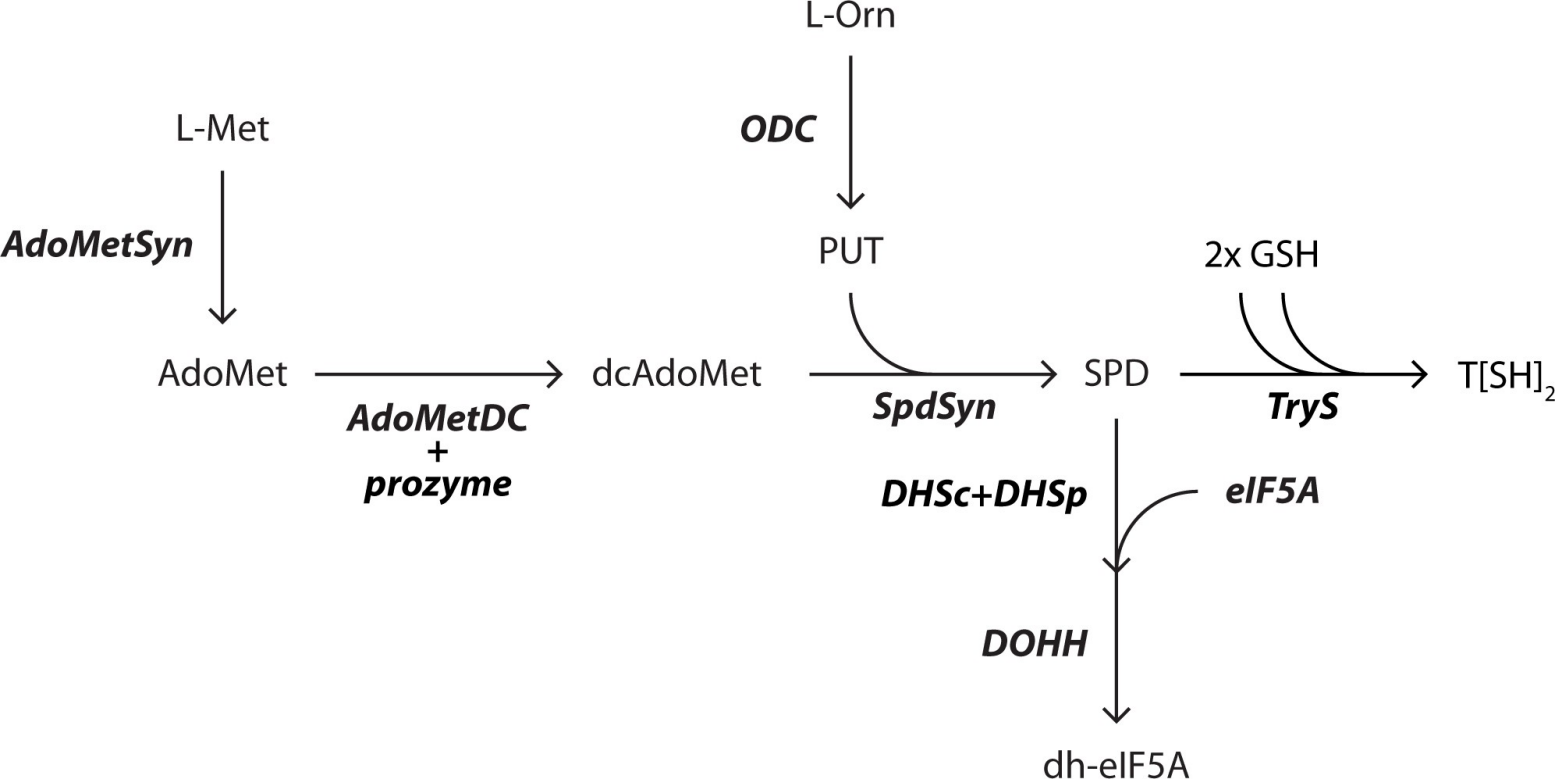
The polyamine biosynthetic pathway in *T*. *brucei*. L-Met, L-methionine; *AdoMetSyn*, *S*-adenosylmethionine synthetase; AdoMet, *S*-adenosylmethionine; *AdoMetDC*, *S*-adenosylmethionine decarboxylase; *prozyme*, AdoMetDC pseudoenzyme required for activity; dcAdoMet, decarboxylated *S*-adenosylmethionine; L-Orn, L-ornithine; *ODC*, ornithine decarboxylase; PUT, putrescine; SPD, spermidine; *SpdSyn*, spermidine synthase; *TryS*, trypanothione synthetase; T[SH]_2_, trypanothione; *DHS*, deoxyhypusine synthase composed of two inactive paralogs in the trpanosomatids *DHSc* and *DHSp*; *DOHH*, deoxyhypusine hydroxylase; *eIF5A*, eukaryotic initiation factor 5A; *dh-eIF5A*, deoxyhypusine eIF5A; GSH, glutathione.

The single-celled eukaryotic parasite *Trypanosoma brucei* is the causative agent of human African trypanosomiasis (HAT), also known as sleeping sickness, and of nagana in cattle. According to the World Health Organization, approximately 65 million people in sub-Saharan Africa are at risk for HAT [1]. Both human infective *T*. *brucei* species (*Trypanosoma brucei gambiense* and *Trypanosoma brucei rhodesiense)* cause a typically fatal disease [2, 3], though the identification of asymptomatic individuals and of parasite reservoirs in the skin suggests individual outcomes are more complicated than previously understood [4, 5]. While vector control and current therapies have contributed to reduced parasite burden over the past 20 years (current cases are <5000 per year), the available drugs are species- and stage-dependent, toxic, and/or difficult to administer [1]. Eflornithine, which is used in combination for the treatment of late stage *T*.*b*. *gambiense* [6, 7], is an irreversible inhibitor of ornithine decarboxylase (ODC), identifying the polyamine biosynthetic pathway (Fig 1) as a validated pathway for the treatment of HAT [8]. In this same pathway, *S*-adenosylmethionine decarboxylase (*Tb*AdoMetDC), which generates the decarboxylated AdoMet (dcAdoMet) necessary for biosynthesis of the polyamine spermidine, was shown to be essential in *T*. *brucei* by genetic studies [9]. Inhibitors of *Tb*AdoMetDC with *in vivo* anti-trypanosomal activity have also been described [10–14].

Polyamines play important cellular roles in transcription and translation [15–17]. Spermidine is essential in all eukaryotes as a substrate for the hypusine modification of translational elongation factor eIF5a, which has a global role in translational elongation and termination [18, 19]. Furthermore, in the trypanosomatids, spermidine plays a specialized role and is conjugated to glutathione to form trypanothione, an essential redox cofactor [7]. In higher organisms, polyamine biosynthesis is tightly regulated and spermidine has been shown to feedback regulate AdoMetDC and ODC at the levels of transcription, translation, and protein stability [15–17]. In mammals and plants, AdoMetDC translation is controlled by an mRNA upstream open reading frame (uORF) that leads to ribosome stalling when spermidine levels are high, and ODC levels are controlled by protein turnover mediated by an inhibitory binding protein termed antizyme [20]. These regulatory mechanisms are absent in *T*. *brucei* [8]. In trypanosomatids, genes are transcribed constitutively in polycistronic units and undergo trans-splicing reactions simultaneously with polyadenylation to generate mature, monocistronic mRNAs [21]. Transcriptional regulation is generally lacking and gene expression is controlled post-transcriptionally by mRNA stability, translational regulation, and protein stability.

We previously reported that *T*. *brucei* AdoMetDC is regulated by a novel allosteric mechanism. In mammals, AdoMetDC is active as a homodimer [22], whereas, in the trypanosomatids, we demonstrated that *Tb*AdoMetDC is functional only as a heterodimer formed between a catalytically impaired AdoMetDC subunit and inactive paralog (pseudoenzyme), we termed prozyme [23–25]. Both *Tb*AdoMetDC and prozyme are essential for enzyme activity and *T*. *brucei* cell viability [9]. Monomeric *Tb*AdoMetDC is inactive due to autoinhibition by its N-terminus [24]. Upon heterodimerization with prozyme, the N-terminal α-helix repositions to the heterodimer interface, relieving the autoinhibition and generating the active enzyme. Furthermore, prozyme also appears to be involved in regulating the polyamine biosynthetic pathway in *T*. *brucei* [9, 26]. Either knockdown or chemical inhibition of *Tb*AdoMetDC led to an increase in prozyme proteins levels suggesting *T*. *brucei* regulates prozyme to compensate for reduced *Tb*AdoMetDC activity [9, 14, 26, 27]. However, the mechanistic basis for how *T*. *brucei* regulates prozyme expression has not been fully elucidated.

Our previous studies suggested that prozyme expression was post-transcriptionally controlled most likely at the level of translation. Levels of *prozyme* mRNA were not changed in response to *Tb*AdoMetDC knockdown or inhibition, but we identified alternatively spliced variants of *prozyme* mRNA showing that the longest mRNA contained a putative secondary structure suggestive of a potential regulatory role in translation [9, 26]. In mammalian cells, spermidine is a key metabolic signal that regulates expression and activity of the polyamine pathway biosynthetic enzymes [15, 17]. However, in *T*. *brucei*, knockdown or inhibition of other pathway enzymes (e.g. *Tb*ODC or spermidine synthase) did not affect prozyme protein levels despite causing cellular concentrations of spermidine and/or putrescine to decrease [28]. Thus, neither putrescine nor spermidine are likely to be involved in regulating prozyme expression. Instead, we found correlative evidence that dcAdoMet might be a regulatory metabolite. Herein, we expand on these findings by demonstrating that prozyme protein levels are controlled translationally, that the *Tb*AdoMetDC protein itself acts as a suppressor of prozyme expression in an enzyme activity-independent manner, and we provide additional evidence that dcAdoMet, the product of AdoMetDC, acts as the key signal in a feed-back regulatory mechanism.

## Results

### The rate of prozyme translation is upregulated upon chemical inhibition of *Tb*AdoMetDC

As noted above prozyme protein levels increase in response to inhibition of *Tb*AdoMetDC with the mechanism-based irreversible inhibitors MDL-73811 or Genz-644131 [9, 26, 27]. In the presence of cycloheximide (CHX), prozyme upregulation was abolished, and endogenous prozyme protein levels were stable for > 6 h, suggesting a translational mechanism [9]. To extend these findings we monitored prozyme translation directly by labeling nascent prozyme protein with deuterated leucine (^2^H_7_-leucine). The abundance of both labeled (heavy ^2^H-leucine) and unlabeled (light ^1^H-leucine) prozyme peptides was then simultaneously determined by mass spectrometry using selected reaction monitoring (SRM). Leucine was chosen as the labeling reagent because preliminary studies showed prozyme protein levels were not affected by changes in leucine concentration (10–25 μM) in the media (S1A Fig), whereas they were impacted by changes in methionine (discussed below). HMI-19 cell culture media contains >800 μM leucine but we established that 10 μM leucine was sufficient to support cell growth (S1B Fig) while maintaining the prozyme regulatory response in cells treated with Genz-644131 (S1A Fig) (leucine concentrations in human blood and cerebral spinal fluid are reported to be 150 and 14 μM, respectively [29]). Leucine concentrations below 10 μM led to reduced cell growth and to reduced ability of cells to upregulate prozyme expression, likely due to the effects of starvation on overall protein synthesis. To prevent the complication of detecting peptides with combinations of heavy and light isotopic leucine, we monitored initially two peptides (SAFPTGHPYLAGPVDR (residues 157–172) and LEGFTVVHR (residues 297–305)) both of which contained only a single leucine, leading to a 7 Da shift in molecular mass per incorporated ^2^H_7_-leucine. Peptide LEGFTVVHR showed a lower limit of detection and was used to monitor ^2^H_7_-leucine incorporation in all subsequent studies.

**Fig 2:**
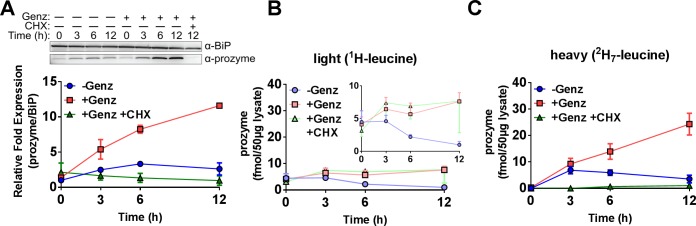
AdoMetDC prozyme translation rates increase in the presence of an AdoMetDC inhibitor. BSF 427 cells were washed and transferred into leucine-free medium supplemented with ^2^H_7_-leucine (10 μM) plus and minus Genz-644131 (Genz) (15 nM) and/or CHX (50 μg/mL) as indicated for 0, 3, 6, and 12 h. (A) Western blot analysis of prozyme and *Tb*BiP (loading control) and the quantitation of prozyme protein levels normalized to *Tb*BiP and to the no Genz control (–Genz) in graph format. (B) Quantitation of unlabeled (^1^H-leucine; light) prozyme per 50 μg total lysate by SRM analysis, where the inset shows the same graph with a subset of the axis range. (C) Quantitation of labeled (^2^H_7_-leucine incorporated; heavy) prozyme per 50 μg total lysate by SRM analysis. Error bars represent SEM of biological replicates, where n = 3 except for +Genz +CHX at 0, 3, and 6 h, where n = 2.

Bloodstream form (BSF) *T*. *brucei* Lister 427 cells were cultured in the presence of ^1^H-leucine (light), washed in PBS, and then transferred to leucine-free medium supplemented with 10 μM ^2^H_7_-leucine (containing dialyzed serum so that ^1^H-leucine was not introduced from the serum). Simultaneously vehicle control (water), Genz-644131, or both Genz-644131 and CHX were also added. Prozyme expression levels were then monitored over 12 h by both Western blot and SRM. Western blot analysis indicated total prozyme protein levels increased in a time-dependent manner for cells treated with Genz-644131 (Fig 2A and S1C Fig), recapitulating our previous results. Prozyme upregulation was abolished, as expected, in cells simultaneously treated with Genz-644131 and CHX. SRM analysis of the unlabeled prozyme peptide (pre-existing ^1^H-leucine prozyme) showed prozyme concentrations were stable over the 12 h time course when treated with Genz-644141, while we observed some turnover of the protein in the absence of Genz-644141 over the 3–12 h time period (Fig 2B and S1 Table). In contrast, we observed a time-dependent increase in ^2^H_7_-leucine-labeled prozyme peptides by SRM analysis in samples treated with Genz-644131, and the rate of this increase was significantly greater than observed for the untreated (minus Genz-644131) control (Fig 2C and S1 Table). Addition of CHX prevented incorporation of ^2^H_7_-leucine into the prozyme peptide, confirming that ^2^H_7_-leucine incorporation was dependent on translation. An increased rate of incorporation was observed in the untreated control at the first time-point (3 h) that can be attributed to a feeding effect as cells were transferred into rich media after the wash step (see below effects of methionine starvation). The rate of incorporation into untreated controls returned to low levels by 6 h, while cells treated with Genz-644131 continued to incorporate ^2^H_7_-leucine at an increased rate throughout the 12 h study. These data show that prozyme translation rates increase when *Tb*AdoMetDC is inhibited with Genz-644131, consistent with a translational regulatory mechanism. They also suggest that stabilization of prozyme from degradation occurs upon treatment with Genz-644131 and may also contribute to the increased pools of prozyme under this condition.

### *Tb*AdoMetDC is a negative regulator of prozyme expression

In our previous work, we sought to determine whether *Tb*AdoMetDC might function to regulate prozyme expression levels by either directly binding to the prozyme mRNA or by interacting with other RNA binding proteins [26]. In that study, we generated a *Tb*AdoMetDC RNAi cell line that expressed human AdoMetDC (*Hs*AdoMetDC) under the control of a tetracycline (Tet) promoter, such that the addition of Tet led to the simultaneous expression of *Hs*AdoMetDC and knockdown of *Tb*AdoMetDC. Expression of *Hs*AdoMetDC led to restoration of WT prozyme levels upon knockdown of *Tb*AdoMetDC. However, based on western blot analysis, we estimated that about 20% of *Tb*AdoMetDC protein remained after RNAi knockdown, and thus this study did not rule out the possibility that *Tb*AdoMetDC protein itself was a negative regulator of prozyme expression.

**Fig 3:**
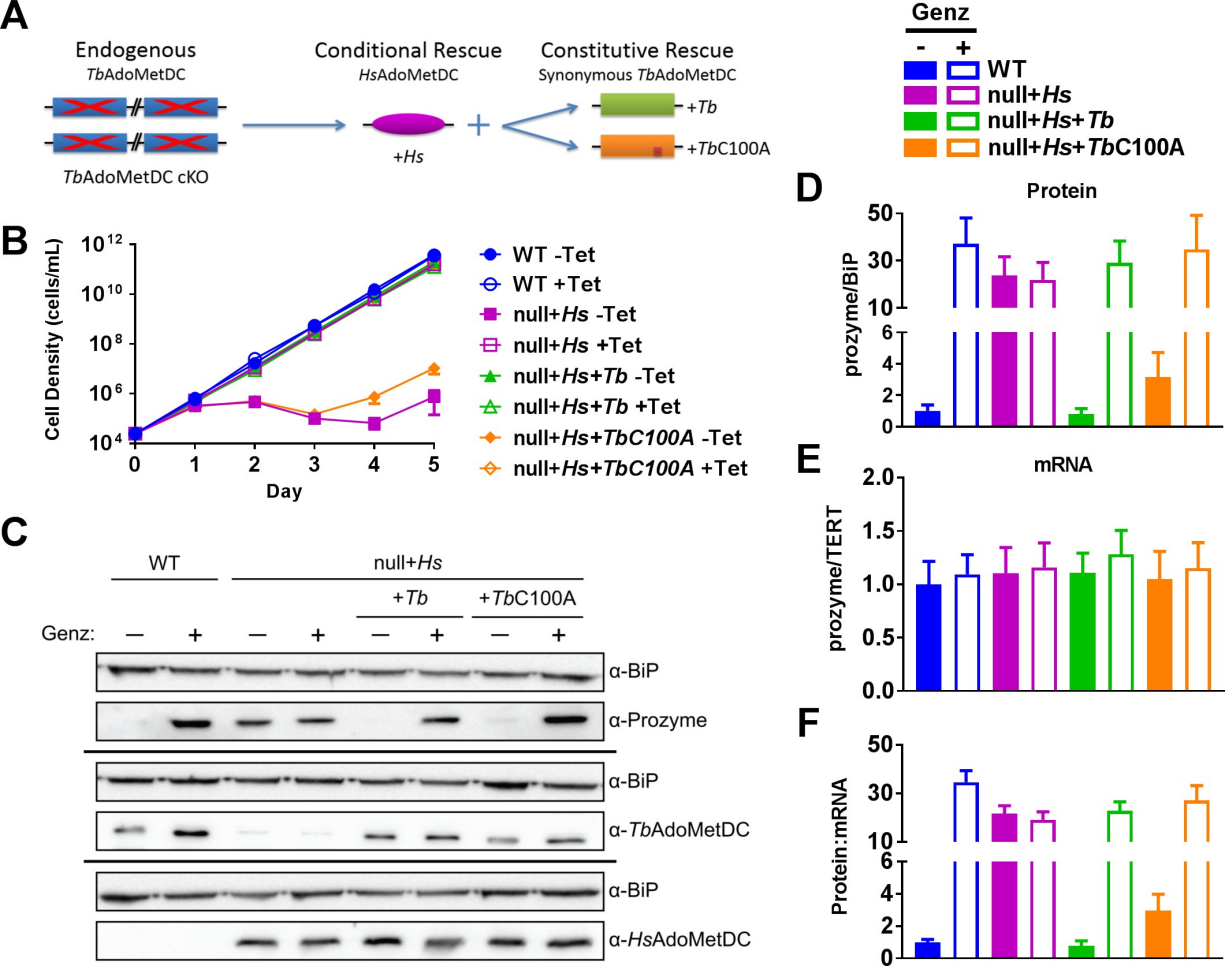
*Tb*AdoMetDC is a negative regulator of prozyme expression. (A) Schematic representation of *Tb*AdoMetDC knockout and complementation strategies. (B) *T*. *brucei* growth analysis of WT SM, *Tb*AdoMetDC null+*Hs*, *Tb*AdoMetDC null+*Hs*+*Tb*, *Tb*AdoMetDC null+*Hs*+*Tb*C100A cell lines ±Tet. Error bars represent SD of three biological replicates. (C) Western blot analysis of WT SM, *Tb*AdoMetDC null+*Hs*, *Tb*AdoMetDC null+*Hs*+*Tb*, *Tb*AdoMetDC null+*Hs*+*Tb*C100A cell lines ±Genz-644131 (24 h) at the respective EC_50_ for each parasite cell line (see S2C Fig). (D) Quantitation of prozyme protein levels in Fig 3C normalized to BiP loading control relative to WT –Genz sample. (E) RT-qPCR analysis of prozyme of WT SM, *Tb*AdoMetDC null+*Hs*, *Tb*AdoMetDC null+*Hs*+*Tb*, *Tb*AdoMetDC null+*Hs*+*Tb*C100A cell lines ±Genz-644131 (24 h). mRNA levels are normalized to TERT expression and the WT–Genz sample. (F) Ratio of prozyme protein levels to mRNA levels from sample samples shown in Fig 3D, 3E, and 3F. Error bars represent SEM of biological replicates, where n = 3 except for null+*Hs*+*Tb*C100A +Tet, where n = 2.

To further address this question, herein we generated a *Tb*AdoMetDC null cell line in the presence of conditionally expressed *Hs*AdoMetDC under control of the Tet promoter (*Tb*AdoMetDC null+*Hs*) (Fig 3A). *T*. *brucei* contains two identical *amd* genes (encoding AdoMetDC) in the genome (Tb427.06.4410 and Tb427.06.4460 in TriTrypDB) [30] and thus as a diploid organism, contains four copies of the gene. To generate the *amd* null cell line, we used the Cre-loxP system [31] to remove the selectable markers after knockout of the first two alleles so that the markers could be reused in the subsequent knockout of the final gene copies (Experimental Procedures). The *Hsamd* gene was inserted into the rRNA locus to complement the loss of *Tb*AdoMetDC prior to removal of the final two *Tbamd* gene copies. In the absence of Tet, *Tb*AdoMetDC null+*Hs* cells undergo a severe growth defect rescued by expression of *Hs*AdoMetDC (Fig 3B and S2A and S2B Fig). As in our previous studies, addition of the AdoMetDC inhibitor Genz-644131 to wild-type (WT) cells led to induction of prozyme levels detected by western blot analysis 24 h after addition of compound (Fig 3C and 3D and S2C and S2D Fig) [27]. In contrast, in the *Tb*AdoMetDC null+*Hs* cell line, we observed constitutively high levels of prozyme in the presence or absence of Genz-644131 (Fig 3C and 3D and S2D Fig). To confirm that the effects on prozyme expression were caused by changes in protein levels and not in mRNA levels, prozyme mRNA levels were evaluated by quantitative reverse transcription PCR (RT-qPCR) (Fig 3E and 3F), demonstrating that prozyme mRNA levels remained constant in both WT and null+*Hs* cell lines with and without Genz-644131. Of note, Genz-644131 is an equally effective inhibitor of both *Tb*AdoMetDC and *Hs*AdoMetDC [11], thus demonstrating that loss of AdoMetDC activity in cells expressing either *Tb*AdoMetDC and *Hs*AdoMetDC is not sufficient on its own to lead to prozyme expression changes. Our data support a mechanism whereby *Tb*AdoMetDC is a negative regulator of prozyme translation. In its absence prozyme is constitutively expressed at higher levels, and prozyme expression is no longer sensitive to inhibition of AdoMetDC activity.

To confirm that the *Tb*AdoMetDC null+*Hs* line remained capable of regulated prozyme expression we rescued the cell line by transfecting it with a constitutively expressed copy of WT *Tb*AdoMetDC (AdoMetDC null+*Hs*+*Tb*) (Fig 3A and S2A Fig). To differentiate between potential regulatory elements in *Tbamd* genetic sequence and amino acid sequence, the DNA sequence of this complement construct was from a construct codon-optimized for *E*. *coli* expression; thus the mRNA was altered while maintaining the amino acid sequence. Cells harboring the WT *Tbamd* complement construct expressed *Tb*AdoMetDC constitutively, leading to restoration of WT growth even in the absence of Tet when human AdoMetDC is no longer expressed (Fig 3B and S2A Fig). WT prozyme protein expression levels were restored in *Tb*AdoMetDC null+*Hs*+*Tb* cells. Moreover, prozyme could be again upregulated with Genz-644131 treatment (Fig 3C and 3D and S2D Fig). Taken together, these results suggest that the *Tb*AdoMetDC protein, and not *Tb*AdoMetDC gene or mRNA was responsible for the regulatory effect on prozyme protein levels.

We next sought to determine whether the *Tb*AdoMetDC protein or its enzymatic function was necessary for suppression of prozyme expression. Using a parallel approach to above we transfected *Tb*AdoMetDC null+*Hs* cells with a catalytically-dead copy of *Tb*AdoMetDC containing a mutation of the catalytic cysteine (C100) to alanine (AdoMetDC null+*Hs*+*Tb*C100A). The C100A mutant of both human and *T*. *cruzi* AdoMetDC were previously shown to have >100-fold reduced activity over the WT enzyme [32, 33]. Consistent with the lack of activity, *Tb*AdoMetDC-C100A was unable to rescue growth in the absence of *Hs*AdoMetDC expression (–Tet) (Fig 3A and 3B and S2A Fig). However, WT prozyme protein levels were restored in this cell line, and this line was capable of Genz-644131-dependent prozyme upregulation, similarly to the *Tb*AdoMetDC null+*Hs*+*Tb* cell line (Fig 3C and 3D and S2D Fig), suggesting that *Tb*AdoMetDC enzymatic function was dispensable for the regulatory effect. Again, prozyme mRNA levels did not significantly vary among lines or stimulatory conditions and the prozyme protein:mRNA ratios mirrored the changes in protein levels (Fig 3E and 3F). Altogether, we conclude *Tb*AdoMetDC protein suppresses translation of prozyme by an enzyme activity-independent mechanism.

### Inhibition of AdoMetDC correlates with changes to dcAdoMet and prozyme protein levels

In mammalian cells, spermidine plays an important role as a negative feedback regulator affecting translation of AdoMetDC mRNA and turnover of ODC [8]. However, in *T*. *brucei*, prozyme protein levels are unaffected by changes in spermidine concentration [28]. Instead, previous studies suggested that dcAdoMet concentration correlated inversely to prozyme expression levels [26]. To provide further support for the hypothesis that dcAdoMet concentration is involved in controlling prozyme protein levels, we analyzed the effects of three independent mechanisms to reduce cellular dcAdoMet levels. These included our previously described use of AdoMetDC inhibitors, plus two new approaches, knockdown of AdoMet synthetase (AdoMetSyn) and methionine starvation.

**Fig 4:**
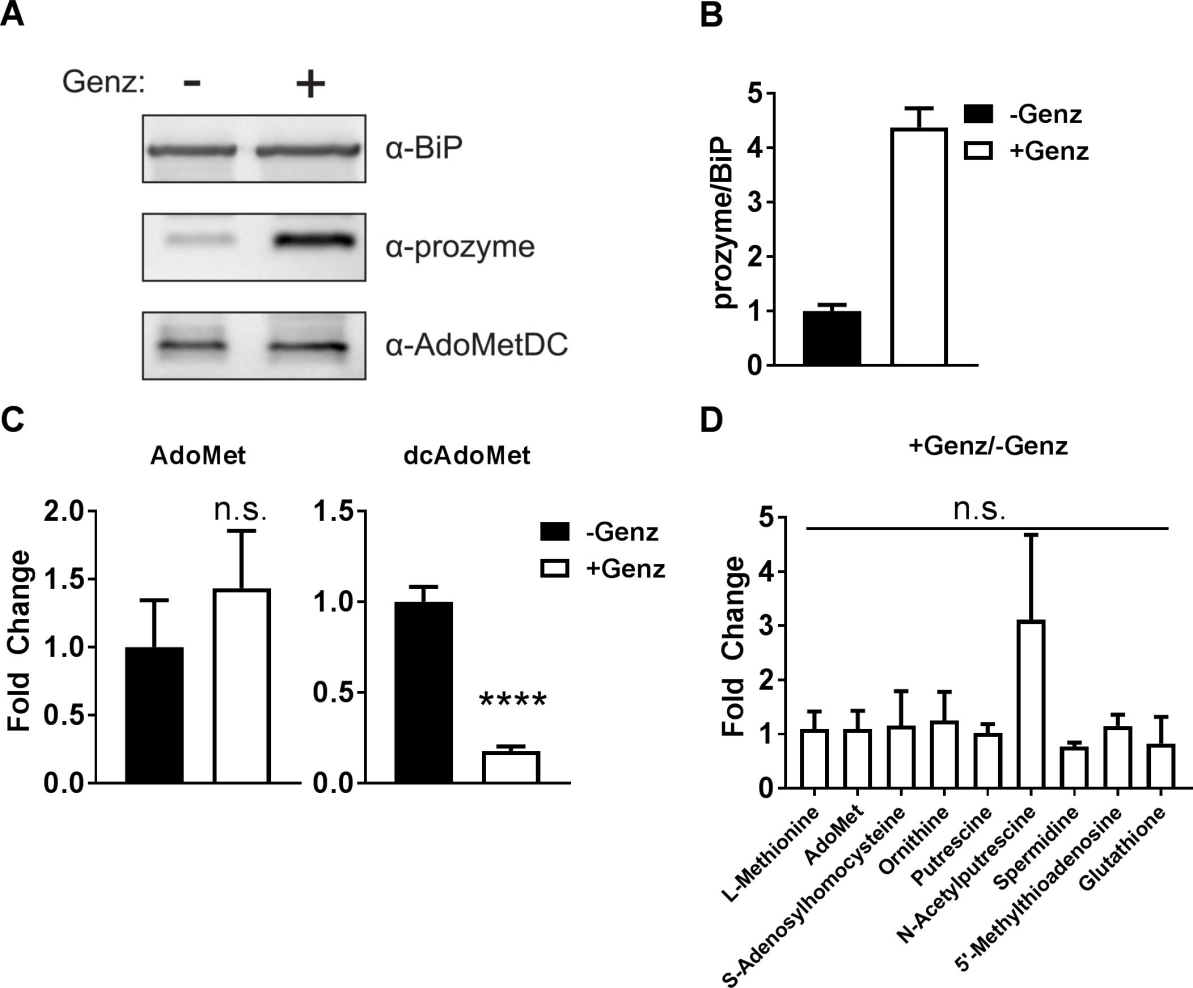
Prozyme protein induction correlates with a depletion of dcAdoMet. BSF 427 cells were treated ±Genz-644131 (15 nM) for 6 h then harvested for western blot and LC-MS analysis. (A) Western blot analysis of prozyme, *Tb*AdoMetDC and *Tb*BiP. (B) Quantitation of prozyme protein levels from Fig 4A, normalized to *Tb*BiP relative to –Genz. (C) AdoMet (left panel) and dcAdoMet (right panel) metabolite levels measured by LC-MS normalized to total protein determined by BCA Assay relative to –Genz. (D) Fold change of selected polyamine pathway metabolites measured by LC-MS normalized to –Genz. Measurements shown in Fig 4B and 4C were taken from the same samples but were performed by separated methods. For Fig 4B, 4C and 4D, error bars represent SEM of three biological replicates where n = 3. For C and D, significance was determined by multiple T test analysis in GraphPad Prism comparing +Genz and –Genz. **** P<0.0001.

We quantitated the relative levels of AdoMet and dcAdoMet in parasites before and after treatment with the AdoMetDC inhibitor Genz-644131 for 6 h. An early time point was chosen so that the results would be independent of effects on cell growth that occur upon more extended incubation. Under conditions where prozyme was upregulated (+Genz) (Fig 4A and 4B and S3 Fig) dcAdoMet pools were depleted by 82% (*p* value <0.0005) (Fig 4C), while AdoMet pools were slightly elevated though this latter change was not statistically significant (Fig 4C). Using a broader targeted metabolomics analysis (112 soluble metabolites) of these same cell extracts we did not identify any other metabolite that significantly changed or correlated with prozyme upregulation (Fig 4D, S2 Table and S4 Fig). AdoMet showed no significant change in this data set (dcAdoMet was not measured). We previously showed that AdoMet levels are ~200-fold higher than dcAdoMet levels in *T*. *brucei*, [28] thus a loss of flux into dcAdoMet would not be expected to impact the AdoMet pools. Polyamine levels were unchanged with the exception of a modest (3.1 ± 1.6)-fold increase in *N*-acetylputrescine in the presence of Genz that was not statistically significant. In previous studies we did observe an increase in putrescine and a decrease in spermidine after a longer time of incubation with MDL 72811 (72 h), but through co-treatment with eflornithine we were able to show that the elevated putrescine levels were not linked to prozyme expression [26]. These current data suggest that *N*-acetylputrescine may be formed to buffer against the accumulation of putrescine. *N*-acetylputrescine has been observed in other published metabolomic studies in *T*. *brucei* [34, 35] and its levels were shown to be affected similarly to putrescine after treatment with eflornithine [35]. The enzyme responsible for its formation and its role in parasite biology are unknown.

### *Tb*AdoMetSyn knockdown upregulates prozyme expression

As a second independent approach to manipulate the cellular dcAdoMet levels we assessed the influence of upstream pathway enzymes and metabolites on prozyme expression. Both AdoMet and dcAdoMet pools are controlled by their biosynthesis. The trypanosomatids including *T*. *brucei* encode a putative AdoMet synthetase (*Tb*AdoMetSyn), which utilizes ATP and methionine to catalyze formation of AdoMet. To validate its function, we expressed and purified recombinant *Tb*AdoMetSyn using affinity His_6_-tag and characterized its activity at varying ATP and methionine concentrations. These studies demonstrated that the *adometsyn* gene indeed encodes an active AdoMetSyn with kinetic parameters similar to those reported for the *Leishmania infantum* enzyme [36]. (Table 1 and S5A Fig).

**Table 1:** Steady-state kinetics analysis of recombinant *Tb*AdoMetSyn.

	k_cat_ (s^-1^)		K_m_ (mM)	
	ATP	Methionine	ATP	Methionine
*Tb*AdoMetSyn (150 nM)	0.21 (0.20–0.23)	0.19 (0.18–0.19)	0.31 (0.24–0.40)	0.031 (0.025–0.039)
*Tb*AdoMetSyn (75 nM)	0.24 (0.22–0.25)	0.21 (0.20–0.22)	0.35 (0.27–0.44)	0.045 (0.034–0.061)

Data were collected in triplicate. Range in parenthesis represents 95% confidence interval. k_cat_ values reported are from respective substrate dose-response curves. Data were collected at two different enzyme concentrations to demonstrate that the V_max_ was linearly dependent on enzyme concentration (k_cat_ = V_max_/[E]).

**Fig 5:**
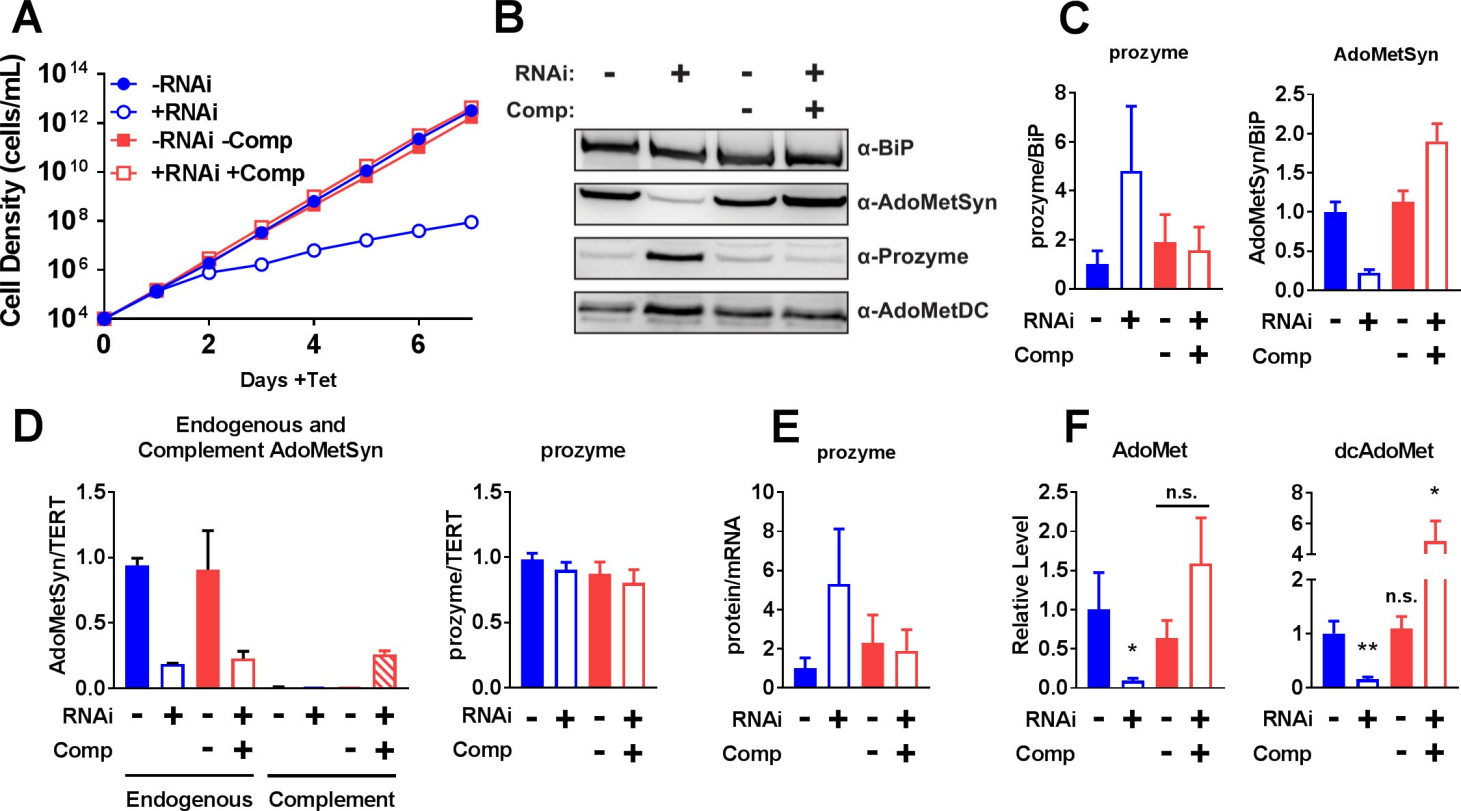
*Tb*AdoMetSyn knockdown upregulates prozyme expression. (A) Growth analysis of *Tb*AdoMetSyn-RNAi (Blue) and *Tb*AdoMetSyn-RNAi+Comp (Red) cell lines ±Tet. Error bars represent SD of three biological replicates, but error bars are smaller than symbols. (B) Western blot analysis of *Tb*AdoMetSyn, prozyme, AdoMetDC and *Tb*BiP ±Tet (48 h). Error bars represent SD for three biological replicates. (C) Quantitation of western blots from Fig 5B prozyme (left panel) and *Tb*AdoMetSyn (right panel) protein levels normalized to *Tb*BiP relative to *Tb*AdoMetSyn-RNAi –Tet *B*. (D) RT-qPCR analysis of *Tb*AdoMetSyn (left panel) and prozyme (right panel) mRNA levels normalized to TERT expression ±Tet (48 h). Endogenous (solid) and complement (striped) *Tb*AdoMetSyn were normalized to endogenous *Tb*AdoMetSyn-RNAi –Tet. Prozyme was normalized to RNAi –Tet. (E) Prozyme protein:mRNA ratio from Fig 5C and 5D. (F) AdoMet (left panel) and dcAdoMet (right panel) metabolite levels ±Tet (48 h) measured by LC-MS/MS relative to *Tb*AdoMetSyn-RNAi –Tet (Blue, filled). For Fig 5C, 5D, 5E and 5F, error bars represent SEM of three biological replicates, n = 3. For F, significance was determined by multiple T test analysis in GraphPad Prism comparing samples to *Tb*AdoMetSyn-RNAi –Tet. * P<0.05, **P<0.01.

We next evaluated the effects of *Tb*AdoMetSyn knockdown on regulation of the polyamine pathway via prozyme protein levels. *T*. *brucei* has 18 identical copies of *Tb*AdoMetSyn arising from a 9-copy tandem array (Tb427.6.4840-Tb427.6.4920) and the diploid genome. Targeting the full array to generate a conditional knockout line would be technically challenging. Instead we generated an RNAi line to study the effects of *Tb*AdoMetSyn knockdown on cell growth, AdoMet and dcAdoMet pools, and prozyme expression. This *Tb*AdoMetSyn RNAi cell line was engineered by inserting a *Tb*AdoMetSyn hairpin sequence (nt 602–1039) under control of the Tet promoter into the ribosomal gene cluster of BSF cells. Addition of Tet to the *Tb*AdoMetSyn RNAi cell line led to a significant growth effect starting at 48 h (Fig 5A). This growth arrest corresponded to an 80% decrease in *Tb*AdoMetSyn protein and mRNA levels at 48 h (Fig 5B–5D and S5B Fig) as evaluated by western blot and RT-qPCR respectively. Concomitant with *Tb*AdoMetSyn knockdown, prozyme protein levels were significantly upregulated 48 h after the addition of Tet (Fig 5B and 5C and S5B Fig).

To demonstrate that the observed effects were caused by *Tb*AdoMetSyn knockdown we transformed the *Tb*AdoMetSyn RNAi line with a RNAi-resistant *T*. *brucei Tb*AdoMetSyn (S6 Fig) expression construct to provide genetic complementation of the knockdown (*Tb*AdoMetSyn RNAi+Comp) (Fig 5A–5D). In this line, *Tb*AdoMetSyn protein levels were similar to WT levels, and WT growth rates were restored (Fig 5A–5C). While we could not distinguish between endogenous *Tb*AdoMetSyn and enzyme expressed from the scrambled complement construct by western blot analysis, RT-qPCR analysis showed that endogenous mRNA was similarly reduced in both the *Tb*AdoMetSyn RNAi and *Tb*AdoMetSyn RNAi+Comp lines (Fig 5D). Prozyme protein levels were also restored to WT levels by genetic complementation of the RNAi line (Fig 5B and 5C). Prozyme mRNA levels do not change significantly under *Tb*AdoMetSyn RNAi or complementation conditions, thus the upregulation of prozyme protein levels upon *Tb*AdoMetSyn knockdown occurs post-transcriptionally (Fig 5D and 5E), similarly to our previous observations upon AdoMetDC knockdown or inhibition.

Finally, we analyzed the effects of *Tb*AdoMetSyn knockdown on AdoMet and dcAdoMet intracellular pools using LC-MS/MS. Knockdown of *Tb*AdoMetSyn led to an 80–90% depletion of both AdoMet and dcAdoMet pools 48 h after the addition of Tet (Fig 5F). Complementation of the *Tb*AdoMetSyn RNAi by the scrambled *Tb*AdoMetSyn rescued construct restored levels of both AdoMet and dcAdoMet to WT levels.

These data provide the first evidence of prozyme upregulation without direct manipulation of AdoMetDC. Furthermore, dcAdoMet levels again correlate inversely with prozyme levels. While AdoMet pools were also reduced after *Tb*AdoMetSyn knockdown, they were not affected by AdoMetDC knockdown or inhibition, and thus are unlikely to play a role in prozyme regulation.

### Methionine starvation upregulates prozyme expression

**Fig 6:**
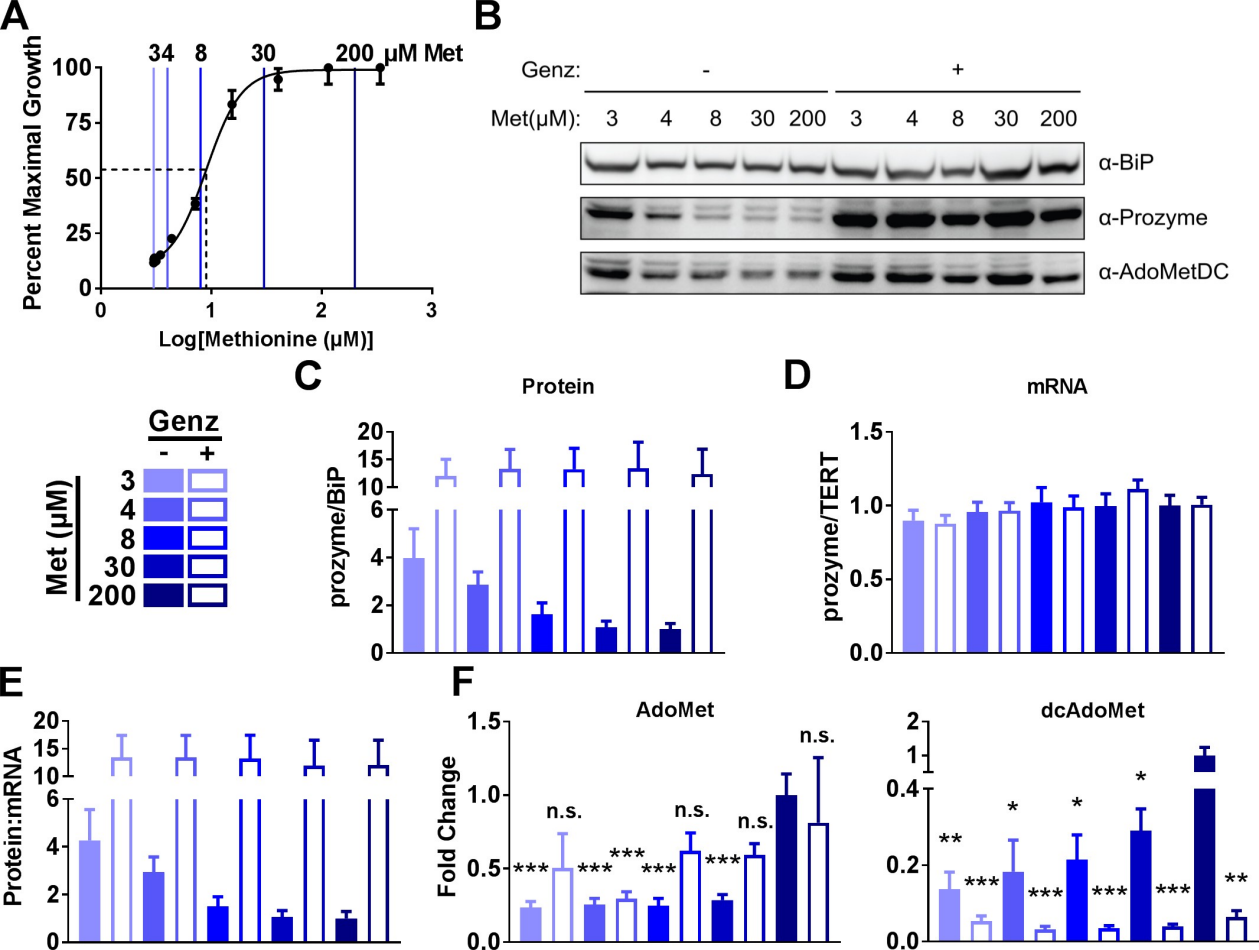
Methionine starvation upregulates prozyme. (A) Cell viability of BSF 427 cells grown for 48 h in methionine-free HMI-19 media with 10% FBS supplemented with varying levels of methionine (Met) as indicated. Viable cells were quantitated by CellTiter Glo assay. Colored bars mark methionine levels used in Fig 6B, 6C, 6D, 6E and 6F. The dotted line marks the fitted EC_50_ (mean ± standard error of the fit = 9.0 ± 0.85 μM). Error bars represent SD for three biological replicates. (B) BSF 427 cells cultured for 48 h with varying levels of methionine and then treated ±Genz-644131 (15 nM) 24 h prior to harvest. (C) Quantitation of the western blot in Fig 6A normalized to Met 200 μM –Genz. (D) RT-qPCR analysis of prozyme mRNA from cultures used in *A* normalized to Met 200 μM –Genz. (E) Protein:mRNA ratio. (F) Fold change of dcAdoMet and AdoMet metabolite levels measured by LC-MS normalized to Met 200 μM –Genz. For Fig 6C, 6D, 6E and 6F, error bars represent SEM for three biological replicates, n = 3. For F, significance was determined by multiple T test analysis in GraphPad Prism comparing samples to Met 200 μM –Genz. * P<0.05, ** P<0.01, *** P<0.005.

Since AdoMet is synthesized from methionine and ATP as third independent approach to reduce dcAdoMet levels we used methionine starvation to manipulate the pathway without directly perturbing the enzyme activity levels. We examined the effects of methionine starvation on cell growth, prozyme expression levels, and AdoMet and dcAdoMet levels. To determine the concentration range that would be appropriate for the methionine starvation study we first measured methionine concentration in FBS (S3 Table) by LC-MS/MS (methionine = 30 ± 1.9 μM, which is similar to that reported for human serum [29]). Thus, in *T*. *brucei* medium (HMI-19) supplemented with 10% FBS, the minimum methionine concentration will be 3 μM, whereas the concentration in standard HMI-19 medium is 200 μM. To determine the minimum methionine levels necessary for BSF 427 *T*. *brucei* cell viability, we performed a methionine dose response study (3–200 μM) where cells were grown for 48 h to determine the minimum methionine levels necessary *T*. *brucei* cell viability. Cells exhibited an increasingly severe growth defect as methionine levels fell below 30 μM (Fig 6A). Relative half-maximal growth rate was at 9.0 (8.2–9.8) μM (95% confidence interval in parenthesis). Finally, we also performed long-term growth rate analysis of cells grown at select methionine levels, and found that although cells grown at 3 μM methionine grow slower, they remain viable (S7A Fig).

The effects of varying methionine concentration on prozyme expression were then assessed to provide orthogonal support for the role of dcAdoMet in prozyme regulation. We observed a methionine dose-dependent upregulation of prozyme for methionine medium concentrations below 4 μM. Prozyme protein levels could be further increased by the addition of Genz-644131 for 24 h at all levels of methionine (Fig 6B, 6C and S7B Fig). RT-qPCR analysis indicated that prozyme mRNA does not change significantly in any of these conditions, demonstrating prozyme expression is regulated post-transcriptionally (Fig 6D). The effects of methionine depletion on prozyme expression do not result simply from nutrient starvation, as depletion of leucine from the growth media did not impact prozyme levels (S1 Fig). Interestingly, we also observed some upregulation of AdoMetDC at low methionine concentrations (Fig 6B). AdoMet and dcAdoMet measurements were made by LC-MS/MS analysis, which revealed that both AdoMet and dcAdoMet pools decreased as methionine was reduced; the concentration of dcAdoMet in cells grown at 3 μM methionine was 95-fold lower than for cells grown in 200 μM (Fig 6F). dcAdoMet levels were further decreased after treatment (24 h) with Genz-644131 (Fig 6F).

Thus we have shown by three independent genetic or chemical methods that depletion of dcAdoMet pools correlates with an upregulation of prozyme, providing further evidence for causal link between prozyme levels and dcAdoMet concentration.

## Discussion

**Fig 7:**
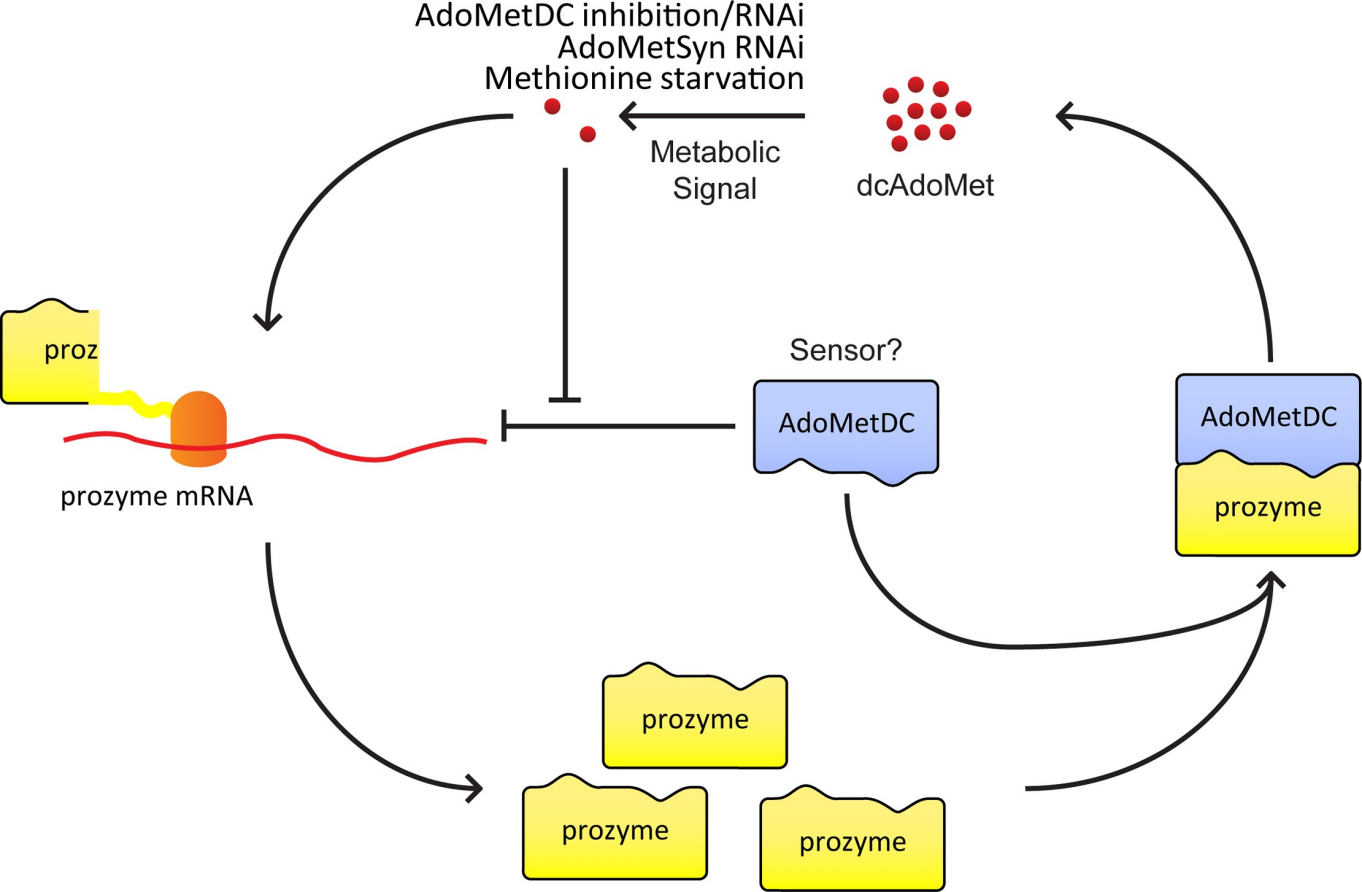
Hypothetical model for regulation of prozyme translation. Monomeric *Tb*AdoMetDC has low activity and active enzyme is generated by heterodimerization with prozyme. The active complex catalyzes the decarboxylation of AdoMet to dcAdoMet. Upon stimuli, including knockdown or chemical inhibition of *Tb*AdoMetDC, knockdown of *Tb*AdoMetSyn, or methionine starvation, dcAdoMet pools decrease. This depletion signals upregulation of *prozyme* mRNA translation. Newly synthesized prozyme then complexes with inactive *Tb*AdoMetDC monomers to form active heterodimers to compensate for the loss of flux in the decarboxylation of AdoMet. *Tb*AdoMetDC protein suppresses *prozyme* mRNA translation when dcAdoMet levels are high. This repression is alleviated by low levels of dcAdoMet.

Polyamine biosynthesis is tightly regulated in many eukaryotes, however the mechanism by which this regulation is achieved is very different in *T*. *brucei* [8]. In contrast to mammalian cells, in *T*. *brucei*, the polyamines spermidine and putrescine do not play significant roles in regulating polyamine biosynthesis in general, or in regulating *Tb*AdoMetDC activity or prozyme expression, specifically [28]. Instead, prozyme regulates *Tb*AdoMetDC at the enzyme level while at the cellular level prozyme protein levels are responsive to perturbations that effect pathway flux (e.g. *Tb*AdoMetDC RNAi or chemical inhibition) [9, 25]. Herein, we have shown that the increase in prozyme protein in the presence of Genz-644131 occurs at the level of translation by directly measuring the rate of prozyme synthesis with stable isotopes. We then expanded on our mechanistic understanding of this regulation by using a *Tb*AdoMetDC null cell line to show that *Tb*AdoMetDC is a suppressor of prozyme translation in an enzyme activity-independent manner. This is the first demonstration of a non-enzymatic regulatory function for AdoMetDC. We also showed strong correlative evidence using three independent methods that low levels of dcAdoMet trigger a relief of this suppression leading to increased prozyme protein levels, thus associating dcAdoMet with a regulatory function. Together, these data suggest that a two-component regulatory system controls prozyme expression; *Tb*AdoMetDC serves as a negative regulator of translation while the cell also senses dcAdoMet levels, such that translational repression is relieved when dcAdoMet levels are low (Fig 7). Enzymatic activity is not required for the *Tb*AdoMetDC regulatory roles.

It is mechanistically unclear how *Tb*AdoMetDC functions as a suppressor of prozyme expression. Because transcription initiation control is absent in kinetoplastids at the level of individual genes, most regulation occurs post-transcriptionally [21]. Several ribosomal profiling studies suggest there is extensive translational regulation [37–39]. In mammalian cells, dihydrofolate reductase (DHFR) and thymidylate synthase (TS) have been shown to autoregulate translation by binding their own mRNA [40–42]. The same mechanism of regulation was shown for *Plasmodium falciparum* DHFR [43]. Analogously, *Tb*AdoMetDC may interact directly with *prozyme* mRNA to control its translation. Alternatively, this interaction may be mediated by a RNA-binding protein (RBP). In *T*. *brucei*, there are over a hundred predicted RBPs, most of which are uncharacterized [44]. In either case, we hypothesize that *Tb*AdoMetDC would form a complex with *prozyme* mRNA and prevent its translation.

The work described herein also provides the first evidence of prozyme regulation independent of changes to *Tb*AdoMetDC and supports a role for the reaction product dcAdoMet in this regulation. Previous studies depended on genetic knockdown or irreversible chemical inhibition of *Tb*AdoMetDC by Genz-644131. Through knockdown of *Tb*AdoMetSyn or methionine starvation, we were able to deplete downstream metabolite pools and upregulate prozyme independently of manipulation of *Tb*AdoMetDC. dcAdoMet levels were substantially decreased after all three perturbations. A broader metabolite analysis was also undertaken at an early time point (6 h) after initiation of Genz-644131 treatment so that the effects on the metabolome could be separated from cell growth changes that occur after longer incubations. The only significantly altered pathway metabolite was dcAdoMet. These data thus support the hypothesis that dcAdoMet acts as a metabolic signal and its depletion triggers increased prozyme protein expression.

AdoMet is the methyl donor for most cellular methylation reactions, including DNA, RNA, and proteins. The ratio of AdoMet to *S*-adenosylhomocysteine has been used as an index for the methylation ability of the cell [45, 46]. We have shown that inhibition of *Tb*AdoMetDC by Genz-644131 did not significantly affect levels of AdoMet, thus alteration of these ratios is unlikely to control prozyme expression. Furthermore, our studies have shown that we can target either downstream or upstream pathways affecting AdoMet to initiate increased prozyme translation. Based on these findings, we conclude that the control of prozyme expression is methylation independent.

We hypothesize that depletion of dcAdoMet pools alleviates suppression of prozyme translation by *Tb*AdoMetDC, but how dcAdoMet pools are sensed remains an open question. We have previously hypothesized that the putative secondary structure located in the 3’UTR of prozyme mRNA may contain a riboswitch-like function by binding dcAdoMet [26]. In bacteria, AdoMet and *S*-adenosylhomocysteine-binding riboswitches have been characterized that regulate translation of methionine and cysteine metabolism [47–51]. While these mechanisms acted through the 5’UTR, in *T*. *brucei*, 3’UTRs of only a few hundred nucleotides can play major roles in regulating mRNA translation and decay [21]. In one potential model, dcAdoMet could be bound by a riboswitch in the prozyme 3’UTR, and this secondary structure can then be bound by *Tb*AdoMetDC or an associated RBP mediator to inhibit translation of prozyme mRNA. Alternatively, dcAdoMet may be bound directly by *Tb*AdoMetDC, which then serves as the sensor to promote binding of itself or another binding partner to *prozyme* mRNA. The C100A-*Tb*AdoMetDC mutation employed in our studies reduces activity but not AdoMet binding, so there remains the possibility that the active site is involved in sensing dcAdoMet levels. Whether there is a difference in translation rates between the larger and shorter *prozyme* ORF-containing mRNA transcripts also remains to be determined.

An intriguing possibility highlighted by our studies is that the paradigm of pseudoenzymes as metabolic regulators will be found in other trypanosomatid pathways, with evolution of these mechanisms perhaps driven by their reliance on post-transcriptional control mechanisms. In recent years, two other enzyme-prozyme complexes have been identified in *T*. *brucei;* deoxyhypusine synthase (DHS) and protein arginine methyltransferase (PRMT1), both of which also require formation of a complex between enzymatically impaired and inactive paralogs (pseudoenzymes) to generate the active enzyme [52, 53]. However, while these pseudoenzyme/enzyme complexes are required for enzyme activity, a regulatory role for these pseudoenzymes in controlling metabolism similar to the prozyme regulatory mechanism seems unlikely as both DHS [52] and PRMT1 [53] exhibit dependent expression such that knockdown of one subunit led to loss of both subunits. This does not preclude other activities by these pseudoenzymes to regulate metabolism in their respective pathways. A growing literature describes diverse roles for paralogous pseudoenzymes functioning as regulators of their respective enzymes in metazoan genomes [54–56]. Given that *T*. *brucei* relies heavily on post-transcriptional mechanisms for gene regulation, the use of pseudoenzymes in regulatory roles may be enriched relative to other organisms.

## Materials and methods

### Gene accession numbers

*T*. *brucei* genomic sequences were obtained from TriTrypDB and gene accession numbers are as follows: *amd* (encodes AdoMetDC*)* Tb927.6.4460/Tb927.6.4410; *prozyme* Tb927.6.4470; *TbAdoMetSyn* Tb927.6.4840-Tb927.6.4920 (9-copy array); *tert* Tb927.11.10190. The accession number for human *amd* is NM_001634.5.

### *T*. *brucei* cultivation and transfection

Experiments were performed using either *Trypanosoma brucei* bloodstream-form (BSF) 427 or single marker (SM) cells that constitutively express the Tet repressor and T7 RNA polymerase (maintained in the presence of Geneticin (G418)) [57]. *T*. *brucei* cells were cultured in HMI-19 medium [58] with 10% fetal bovine serum (FBS) (Tet-free, heat-inactivated; Gemini Bio-Products) or dialyzed FBS (Tet-free, heat-inactivated; Gemini Bio-Products) at 37°C and 5% CO_2_ [9, 59]. Parasite transfections were performed as previously described [60]. Antibiotics were used at the following concentrations: G418 (2.5 μg/mL; Life Technologies), phleomycin (2.5 μg/mL; InvivoGen), hygromycin (1–2.5 μg/mL; Gemini Bio-Products), puromycin (1 μg/mL; Sigma), tetracycline (Tet) (1 μg/mL; Sigma), and ganciclovir (GCV) (InvivoGen) was used at 40 μg/mL. Genz-644131 (a generous gift from Genzyme, presently Sanofi) was used at 15 nM (10X EC_50_) [27]. Cycloheximide (Sigma) was used at 50 μg/mL [9]. For ^2^H_7_-leucine labeling conditions, cell lines were plated into leucine-free HMI-19 medium (prepared with custom-ordered leucine-free IMDM (Invitrogen)) with 10% dialyzed FBS and supplemented with either 10 μM ^2^H_7_-leucine for SRM analysis or for growth studies supplemented with sterile-filtered leucine (Sigma) dissolved in ddH_2_O to the desired concentrations. For methionine-limiting conditions, cell lines were cultured in methionine-free HMI-19 medium (prepared from custom-ordered methionine-free IMDM (Invitrogen)) supplemented with 10% FBS. Sterile-filtered methionine (Sigma) dissolved in ddH_2_O was then used to supplement medium at desired concentrations. Normal HMI-19 medium contains 200 μM methionine and 800 μM leucine [61], both in about 10-fold excess of concentrations observed in human serum [29].

### Generation of *T*. *brucei amd* null cell lines complemented with human AdoMetDC

PCR reactions were performed with Phusion high-fidelity DNA polymerase (NEB). Plasmids propagated using Stellar (Clontech Laboratories) or Invitrogen One Shot TOP10 (Thermo Fisher Scientific) cells. *T*. *brucei* contains two identical *amd* genes (*Tb*427.06.4410 and *Tb*427.06.4460 in TriTrypDB) that encode AdoMetDC [30], thus as a diploid organism four *amd* genes are present in the genome. Due to limiting availability of resistance markers, knockout (KO) of the four gene copies was performed in two rounds using selectable marker cassettes flanked by loxP sites. Marker cassettes were removed with Cre-recombinase after each round and subsequently reused in following steps as described [31]. The human *amd* Tet-regulated complement construct was inserted into the rRNA locus after removal of the first two alleles. Cloning primers are provided in S4 Table. Starting from SM cells, the first two *Tbamd* loci were replaced with resistance marker cassettes (hygromycin-resistance gene *hyg* and puromycin-resistance gene *pac*) fused to the *Herpes simplex* virus thymidine kinase gene (HSV*tk*), flanked by loxP sites. Resistance markers fused to HSV*tk* were amplified from pHJ17 (*hyg*) and pHJ18 (*pac*) [31] (Addgene) with primers p1/p2 (*hyg* and *pac*). 5’ and 3’ flanking regions of *Tbamd* were amplified from SM genomic DNA with primers p3/p4 (5’UTR–1) and p5/p6 (3’UTR–1). The first pair of KO constructs were generated by fusion PCR of *hyg* or *pac* and 5’UTR–1 and 3’UTR–1 flanking amplicons with primers p7/p8 as described [62] and the resulting PCR fragments cloned into pCR-Blunt II-TOPO vector using Zero Blunt TOPO PCR cloning kit (Thermo Fisher Scientific) (KO1-*hyg*-TOPO and KO1-*pac*-TOPO). Knockouts were performed by concurrent transfection of SM cells with NsiI-excised KO1-*hyg*-TOPO and KO1-*pac*-TOPO under hygromycin and puromycin selection (*Tb*AdoMetDC KO1-*hyg*/*pac*). To recycle the selection markers, a *Tb*AdoMetDC KO1-*hyg*/Pac line was transiently transfected with pLew100cre-del-tetO (Addgene) derived from the construct pLEW100cre by deleting the Tet operator [63] to express Cre recombinase. Transfectants were subjected to negative selection with ganciclovir (40 μg/mL) in the absence of hygromycin and puromycin to select for lines with HSV*tk* excised by Cre recombinase (KO1). The resulting *Tb*AdoMetDC KO1 cell line lacks two of four *amd* alleles. *Hsamd* was cloned from the previously described pET28b-derived plasmid [64] with primers p9/p10 into pLew100v5 [57] under control of a Tet-regulatable promoter (p100-*Hs*AdoMetDC). The sequence was confirmed with primers p33/p34. *Tb*AdoMetDC KO1 cells were transfected with NotI–linearized p100-*Hs*AdoMetDC under selection with phleomycin (*Tb*AdoMetDC null1+*Hs*). The second set of 5’ and 3’ flanking regions of *Tb amd* (internal to the first KO) were amplified from SM genomic DNA with primers p11/p12 (5’UTR–2) and p13/p14 (3’UTR–2) and the KO constructs were generated by fusion PCR with *hyg* or *pac* resistance markers and 5’UTR–2 and 3’UTR–2 flanking amplicons with primers p15/p16 (KO2-*hyg* and KO2-*p*) as described above. The *Tbamd* null line was then generated by sequential transfection of a *Tb*AdoMetDC null1+*Hs* with NsiI-excised KO2-*hyg*-TOPO under hygromycin selection (*Tb*AdoMetDC null2+*Hs*-*hyg*) and then NsiI-excised KO2-*pac*-TOPO under hygromycin and puromycin selection in the presence of Tet (*Tb*AdoMetDC null2+*Hs*-*hyg*/*pac*) and other maintenance antibiotics (G418 and phleomycin). The *hyg* and *pac* resistance genes were then removed under negative selection with GCV in the presence of Tet yielding the final *Tbamd* null cell line that expressed human AdoMetDC under the control of the Tet promoter (*Tb*AdoMetDC null+*Hs*). The absence of *Tbamd* was verified by RT-qPCR (S2B Fig) with primers p45/p46 relative to α–Tubulin with primers p47/p48.

### Complementation of *Tb*AdoMetDC null+*Hs* with *Tbamd*

*Tb*AdoMetDC null+*Hs* was complemented with WT or catalytically-dead *Tbamd*. The DNA sequence that was used had been codon-optimized for *E*. *coli* expression thus this allowed us to introduce a different mRNA sequence while maintaining the amino acid sequence. The catalytically-dead *Tb*AdoMetDC mutant was generated by site-directed mutagenesis of our previously described *E*. *coli Tb*AdoMetDC expression construct [24] subcloned into the pCR-Blunt II-TOPO vector. Primers p21/22 were used to convert the catalytic C100A with PfuTurbo DNA polymerase (Agilent Technologies) (TOPO-AdoMetDCscrm-C100A). The reaction was digested with DpnI (NEB) and transfected into TOP10 cells. WT *Tb*AdoMetDCscrm and catalytically-dead *Tb*AdoMetDCscrm-C100A were amplified with primers p19/20 using pET28bSmt3-*Tb*AdoMetDC [24] and TOPO-AdoMetDCscrm-C100A plasmids, respectively, as templates. PCR products were cloned into the HindIII/BamHI sites of pLew90 for constitutive expression in *T*. *brucei* [57]. Sequences were confirmed with primers p31/p32. *Tb*AdoMetDC null+*Hs* was transfected with NotI-linearized p90-*Tb*AdoMetDCscrm or p90-*Tb*AdoMetDCscrm-C100A under selection with hygromycin (*Tb*AdoMetDC null+*Hs*+*Tb* and *Tb*AdoMetDC null+*Hs*+*Tb*C100A). Incorporation of the *Tbamd* genes were validated by western blot analysis (S2A Fig).

### Cell viability growth assays

Cell growth analyses were performed as previously described using the CellTiter-Glo reagent (Promega) [27]. Determination of the 50% growth inhibitory concentration (EC_50_) of Genz-644131 in *Tb*AdoMetDC null lines was made after 24 h of incubation with drug from a starting inoculum of 1 × 10^5^ cells/mL in HMI-19 with 10% FBS using serial dilutions of Genz-644131 at 0.1% (v/v) DMSO. The leucine and methionine concentration required for 50% maximal growth (EC_50_) was measured in BSF 427 cells after 24 h and 48 h, respectively, from a starting inoculum of 1 × 10^5^ and 3 × 10^3^ cells/mL in HMI-19 prepared as described above.

### Generation of *Tb*AdoMetSyn-RNAi and rescue constructs

Cloning primers are described in Table S4. RNAi target sequences were chosen based on RNAit [65] and primers (Sigma) were designed to amplify *Tbadometsyn* nucleotides 602–1039 (*Tb*AdoMetSyn-RNAi-insert). The insert was amplified by Platinum Taq DNA Polymerase (Invitrogen) and cloned into the pCR8/GW/TOPO vector. Sequencing with primer M13-21 was used to identify a clone with the ORF integrated in the forward direction (TOPO-*Tb*AdoMetSyn-RNAi) and this clone was then inserted into the pTrypRNAiGateway [66] vector by recombination using Gateway LR Clonase II Enzyme mix (Invitrogen), generating a Tet-regulated short-hairpin with the (pTRG-*Tb*AdoMetSyn). The integrity of the insert in the resultant clone was confirmed by sequencing using primers p29/30. SM cells (maintained in G418 1 μg/mL) were transfected with NotI–linearized pTRG-*Tbadometsyn* under selection with phleomycin (*Tb*AdoMetSyn RNAi). To generate an RNAi-resistant complement gene, the *Tbadometsyn* ORF (nucleotides 601–1035) was synthesized by GenScript to contain scrambled codons (different RNA sequence that maintained the correct amino acid sequence (pUC57-*Tb*AdoMetSyn; full sequence in S6 Fig). The *Tbadometsyn* ORF was amplified from this vector using primers p25/26 and inserted into HindIII/BamHI-digested pLew100v5-*hyg*, a modified pLew100v5 vector (gift of George Cross) that contains the *hyg* resistance cassette [57], using the InFusion cloning kit (Takara) (p100H-*Tb*AdoMetSyncomp). Sequences were confirmed with primers p33/p34. *Tb*AdoMetSyn RNAi cells were then transfected with NotI–linearized p100H-*Tb*AdoMetSyncomp under hygromycin selection (*Tb*AdoMetSyn RNAi + comp).

### Cloning, heterologous expression, purification, and enzymatic assay of *Tb*AdoMetSyn

To generate an *E*. *coli* expression construct for *TbAdoMetSyn*, pUC57-*Tb*AdoMetSyn was used as a template, the ORF was amplified using primers p27/28 and inserted into a BamHI/XhoI-digested pET28bTEV plasmid (pET28b (Novagen) plasmid with Tobacco Etch Virus (TEV) protease site substituted for the thrombin site, described in [67] with InFusion cloning kit (Takara) allowing for expression of a His_6_-tagged *Tb*AdoMetSyn. The sequence was verified with primers p35/36 (pET28bTEV-*Tb*AdoMetSyn).

For protein expression, pET28bTEV-*Tb*AdoMetSyn was transformed into Novagen BL21(DE3) cells under kanamycin selection (50 μg/ml) (NEB). Cells were grown at 37°C for 2 h until OD_600_ = 0.4 and cooled to 16°C. After 0.5 h at OD_600_ = 0.6, His_6_-*Tb*AdoMetSyn expression was induced with IPTG (0.2 mM) for 22 h. Cells were pelleted by centrifugation at 3,500 × g for 20 min and resuspended in lysis Buffer A (100 mM HEPES, pH 8.0, 300 mM KCl, 5 mM MgSO_4_, 5 mM imidazole, 10% glycerol (v/v), 0.1% (v/v) triton X-100 and supplemented with 1 mM β-mercaptoethanol, 2 mM phenylmethylsulfonyl fluoride (PMSF), 1 μg/mL leupeptin, 2 μg/mL antipain, 10 μg/mL benzamidine, 1 μg/mL pepstain, and 1 μg/mL chymostatin. Cells were lysed by cell disruption using an EmulsiFlex-C5 (Avestin) at 5,000–10,000 psi, and cell debris was pelleted by centrifugation (50,000 × g for 90 min). Soluble protein was purified from lysate by Ni^2+^-affinity chromatography (HisTrap FF column, GE Healthcare) on an ÄKTA purifier system (GE Healthcare) with Buffer A and Buffer B (Buffer A with 500 mM imidazole). Contaminants were washed off the column with 8% Buffer B and *Tb*AdoMetSyn was eluted with a linear gradient 8–50% Buffer B. *Tb*AdoMetSyn-containing fractions were pooled and imidazole content reduced by 100-fold through serial concentration (Amicon Ultra-15 Ultracell 30K centrifugal filters (Merck Millipore)) and dilution with Buffer A. The His_6_-tag was removed by incubation with 50 μg TurboTEV protease (BioVision) for 16 h. Untagged *Tb*AdoMetSyn was purified by passage through a Ni^2+^-affinity HisTrap FF column and collected in the flow-through. *Tb*AdoMetSyn-containing fractions were pooled and concentrated as above. Protein concentration was determined using Bio-Rad Protein Assay Dye reagent and protein was >95% pure based on SDS-PAGE analysis.

Activity was measured using a previously described spectroscopic assay [68]. Pyrophosphate (PP_i_) release by *Tb*AdoMetSyn was measured with a coupled enzyme system in Pyrophophate Reagent (Sigma) containing a PP_i_-dependent fructose-6-phosphate kinase, aldolase, triosephosphate isomerase, glycerophosphate dehydrogenase. The assay was performed in Assay buffer (50 mM HEPES, pH 8.0, 100 mM KCl, 5 mM MgSO_4_, 2 mM 1,4-dithiothreitol (DTT, Sigma), 0.05% (v/v) Triton X-100 reduced) in in 96-well half-area UV-Star plates (Phenix) with 50 μL Assay buffer, 35 μL Pyrophosphate Reagent, 5 μL ATP (100 mM or 2-fold serial dilutions thereof, Sigma), 5 μL methionine (100 mM or 2-fold serial dilutions thereof, Sigma), and 5 μL purified enzyme (3 μM or 1.5 μM, total volume 100 μL). Absorbance at 340 nm was measured continuously on a Synergy H1 plate reader (BioTek) at 37°C. Rate was determined from the linear fit to the data collected over 10 min. Steady-state kinetic constants (K_m_ and k_cat_) were determined by fitting substrate versus velocity data to the Michaelis-Menten equation in GraphPad Prism.

### Purification of RNA from cells

RNA was purified as previously reported [26]. Briefly, cells (≥5 × 10^7^) were washed 3x with 10 mL of PBS (10mM Na_2_HPO_4_, 1.9 mM KH_2_PO_4_, 137 mM NaCl, 2.7 mM KCl, pH 7.4), resuspended in 100 μL PBS, and 1 mL of Trizol (Life Technologies/Invitrogen) was added. Samples were incubated at RT for 5 min. For long-term storage, samples were flash flash-frozen in liquid N_2_. Samples were extracted with chloroform and purified using RNeasy RNA Purification Kit (Qiagen) per manufacturer's protocol. Total RNA was quantified by measuring OD at 260/280 nm.

### cDNA synthesis and qPCR analysis

Primers for qPCR are listed in S4 Table. cDNA was prepared as previously reported [26]. Briefly, 2 μg RNA was treated with DNaseI (Invitrogen) and quenched with EDTA. cDNA was synthesized with random hexamers (Invitrogen) using Moloney Murine Leukemia Virus Reverse Transcriptase (M-MLV RT) (Invitrogen). cDNA levels were quantified using iTaq Universal SYBR Green Supermix (Bio-Rad) on a CFX 96 (Bio-Rad) or QuantStudio 7 Flex (Applied Biosystems) with a standard curve on each run for each primer. Relative mRNA levels were determined using the Pfaffle method [69] and Telomerase Reverse Transcriptase (TERT) was used as the reference gene [70]. For the *Tb*AdoMetDC null+*Hs* line, α-Tubulin was used as the reference gene.

### Western blot analysis

Cells (10^7^−10^8^) were pelleted by centrifugation (2,000 × g, 5 min), washed 2x with PBS (137 mM NaCl, 2.7 mM KCl, 10 mM Na_2_HPO_4_, 1.8 mM KH_2_PO_4_, pH 7.4), resuspended in 30–50 μL lysis buffer (50 mM HEPES, 100mM NaCl, pH 8.0, freshly supplemented with 5mM β-mercaptoethanol, 2mM PMSF, 1 μg/mL leupeptin, 2 μg/mL antipain, 10 μg/mL benzamidine, 1 μg/mL pepstain, and 1 μg/mL chymostatin) and lysed by 3 freeze/thaw cycles using liquid nitrogen. Cell debris was pelleted by centrifugation at 4°C, supernatant collected and protein concentration quantitated using Bio-Rad Protein Assay reagent. Samples (30 μg per lane) were separated by SDS-PAGE on a 12% gel and transferred to PVDF using the iBlot transfer system (Invitrogen), program P3. Membranes were blocked using 5% Blotting-Grade Blocker (Bio-rad) in TBST (50 mM Tris, 150 mM NaCl, pH 7.4, 0.1% (v/v) Tween-20) followed by incubation with primary antibody overnight at 4°C. After washing 3x with PBS (10 mL, 10 min), membranes were incubated with secondary antibody, either Protein A conjugated to HRP (1:1000, AbCam) or goat α-rabbit conjugated to HRP (Sigma) for 30 min at RT. Membranes were washed 3x with PBS, Supersignal West Pico substrate (ThermoFisher) was added and signal was imaged on a LAS 4000 imager (GE Healthcare). Quantification of western blots was performed using ImageQuant TL 8.1 (GE Healthcare). α-*Tb*AdoMetDC, α-prozyme, and α-*Hs*AdoMetDC antibodies have been previously described [26, 34, 71]. Antibody dilutions used were as follows: α-*Tb*AdoMetDC (rabbit, polyclonal, 1:2000), α-prozyme (rabbit, polyclonal, 1:2000), α-BiP (1:50000, BiP), α-*Hs*AdoMetDC (rabbit, polyclonal, 1:2000, a gift from David Feith), α-*Tb*AdoMetSyn (rabbit, polyclonal, 1: 2000).

### Metabolite analysis

Cells (2 × 10^7^ per sample) were harvested in extraction buffer (80% MeOH, 0.1% formic acid for AdoMet and dcAdoMet targeted analysis and 80% methanol (MeOH) only for broad metabolite analysis) and subjected to 5 freeze/thaw cycles. Cell debris was removed by centrifugation (>17,000 × g, 10 min, 4°C), and the supernatant dried by vacuum centrifuge. For broad metabolite analysis, LC-MS/MS was performed as previously described [72] to provide analysis of 112 metabolites, excluding dcAdoMet. For dcAdoMet and AdoMet targeted analysis, samples were suspended in 150 μL solvent identical to the starting conditions of the chromatography method. Insoluble material was removed by centrifugation (>17,000 × g, 10 min, 4°C) and 10 μL of sample was injected for analysis. A Shimadzu Nexera X2 high-performance liquid chromatography (HPLC) coupled to a SCIEX 6500+ QTRAP was used for quantification of metabolites. Separation of metabolites was performed on a hydrophilic liquid chromatography column (Luna HILIC, 100 x 4.6 mm, 3 μm, 4 Å, Phenomenex). The chromatography gradient consisted of two solvents: A: H_2_O, 0.2% formic acid, 5 mM ammonium acetate, B: 90% acetonitrile, 0.2% formic acid, 5 mM ammonium acetate. Optimal separation and detection was achieved with a flow rate of 1.0 mL/min and by the following gradient: 0.1–2 min 70% B, 2–3 min 20% B, 3–5 min 20% B, 5–5.1 70% B, 5.1–7 min 70% B. Infusion optimization was performed using standards obtained commercially (Affymetrix) or enzymatically synthesized as previously described to obtain optimal precursor and product ion masses for each metabolite [26]. In positive mode, multiple reaction monitoring (MRM) was used for detection and quantification of metabolites. The optimal linear response range of both dcAdoMet and AdoMet was determined using the authentic standards. At least two of the most abundant product ions were monitored and the calculated peak areas were normalized to uridine monophosphate as a spiked internal standard and the amount of total protein in the extracted pellet determined by bicinchoninic acid (BCA) assay. The relative abundance of each metabolite was determined by normalization of dcAdoMet and AdoMet signals to the untreated control. The following pairs of precursor/product ions were monitored: AdoMet (399/250, 399/136) and dcAdoMet (355/250, 355/298, 355/136). To measure methionine levels in FBS (Gemini Bio-Products, Lot A29F), extracts were prepared with 200 μL 100% MeOH per 100μL serum and vortexed vigorously to precipitate protein. Insoluble material was removed by centrifugation (>17,000 × g, 10 min, 4°C) and the supernatant dried by vacuum centrifuge. LC-MS/MS analysis of methionine from FBS was performed as previously described [73]. The following pairs of precursor/product ions were monitored: Methionine (150/104, 150/133).

### Selected reaction monitoring analysis

Cells were cultured at 1 × 10^6^ cells/mL in leucine-free HMI-19 medium with 10% dialyzed FBS and supplemented with 10 μM iso-propyl-d_7_ (((CD_3_)_2_CDCH_2_CH(NH_2_)COOH), ^2^H_7_-leucine) (CDN Isotopes), determined as the minimum L-leucine required to maintain prozyme upregulation by Genz-644131 (S1B Fig). Cells (10^7^−10^8^) were harvested and processed for western blot analysis as above. Samples (50 μg per lane) were separated by SDS-PAGE on a 4–20% gradient precast gel (Bio-Rad). The gel was then stained with GelCode Blue Stain Reagent (ThermoFisher) and a 10 mm slice of the lane centered around 37 kDa was analyzed for unlabeled and d_7_-labeled prozyme by selected reaction monitoring (SRM). The tryptic peptide sequences chosen for analysis were SAFPTGHPYLAGPVDR (residues 157–172) and LEGFTVVHR (residues 297–305). These peptides were chosen because they contain only one leucine each, eliminating any complication from peptides that might potentially have a mix of heavy and light leucine. Additionally, we avoided peptides that were prone to missed cleavages (consecutive R or K, for example), peptides that contained methionine (potential oxidation), and peptides that contained cysteine (potential for incomplete carbamidomethylation). After preliminary studies we settled on LEGFTVVHR (residues 297–305) for quantification due to its lower limit of detection. Stable heavy-isotope-labeled peptides were synthesized as standards by 21st Century Biochemicals with purities of >97% as determined by HPLC. All peptides were synthesized with a C-terminal [^13^C_6_,^15^N_4_] arginine, and were used without further purification. Protein gel pieces were reduced and alkylated with DTT (20 mM) and iodoacetamide (27.5 mM). A sufficient volume of 0.05 μg/μL solution of trypsin (Pierce) in 50 mM triethylammonium bicarbonate (TEAB) was added to completely cover the gel. The gel was allowed to sit on ice for 30 min and then 50 μL of 50 mM TEAB was added and the proteins were digested overnight. Peptides were then extracted from the gel and dried. Samples were reconstituted, spiked with 100 fmol of each heavy-isotope labeled peptide, and solid-phase extraction was performed with an Oasis HLB μelution plate (Waters). Samples were dried and reconstituted in 10 μL of 2% (v/v) acetonitrile (ACN) and 0.1% trifluoroacetic acid in water for SRM analysis. The top seven transitions for each heavy-labeled peptide were determined by monitoring peak areas for all singly and doubly charged b and y ions below m/z = 1,250 and for all doubly and triply charged peptide ions below m/z = 1,000, for a mix of the heavy-labeled peptide standards. These data were analyzed using Skyline v4.1 (http://skyline.maccosslab.org) [74], and collision energies were optimized by a subsequent sample injection. Transitions that had interference from impurities or noise peaks were not included when performing peptide quantifications. Spiked samples were separated on a Dionex Acclaim PepMap100 reverse-phase C18 column (75 μm × 15 cm) using an Ultimate 3000 RSLCnano HPLC system. The HPLC was controlled using Chromeleon Xpress (version 6.8 SR10) and Dionex Chromatography MS Link v. 2.12. Separation of peptides was carried out at 200 nL/min using a gradient from 0%–25% B for 15 min, 25%–35% B for 5 min, and 35%–80% B for 5 min, where mobile phase A was 2% ACN, 0.1% formic acid in water and mobile phase B was 80% ACN, 10% trifluoroethanol, 10% H2O, and 0.1% formic acid. Mass spectrometric analysis was performed on an AB Sciex 6500 QTRAP mass spectrometer in positive-ion low-mass mode, using a NanoSpray III source with a New Objective precut 360 μ PicoTip emitter (FS360-20-10-N20-10.5CT). The source settings were as follows: curtain gas = 30, ion spray voltage = 2,450, ion source gas 1 = 6. Analyst Software v.1.6 was used to run the mass spectrometer. SRM data were analyzed using Skyline v4.1.

## Supporting information

S1 Fig (A) Western blot analysis of BSF 427 cells incubated in leucine-free HMI-19 with 10% dFBS supplemented at varying levels of leucine in the presence or absence of Genz-644131 (15 nM) for 6 h. (B) Cell viability of BSF 427 cells grown for 48 h in leucine-free HMI-19 media with 10% FBS supplemented with varying levels of leucine as indicated. Viable cells were quantitated by CellTiter Glo assay. Data were analyzed in GraphPad Prism to determine the effective concentration at 50% growth (EC_50_). Error bars represent SD for three biological replicates. (C) Replicates of western blot analysis in Fig 2A.
(TIF)

S2 Fig (A) Western blot analysis against *Tb*BiP, *Tb*AdoMetDC, and *Hs*AdoMetDC of WT SM, *Tb*AdoMetDC null+*Hs*, *Tb*AdoMetDC null+*Hs*+*Tb*, *Tb*AdoMetDC null+*Hs*+*Tb*C100A cell lines cultured ±Tet for 48 h to show *Tb*AdoMetDC and *Hs*AdoMetDC protein levels during growth curve in Fig 3B. (B) RT-qPCR analysis of *Tb*AdoMetDC mRNA from WT SM and *Tb*AdoMetDC null+*Hs* normalized to α-Tubulin (C) Genz-644131 dose-response analysis of WT SM, *Tb*AdoMetDC null+*Hs*, *Tb*AdoMetDC null+*Hs*+*Tb*, *Tb*AdoMetDC null+*Hs*+*Tb*C100A cell lines incubated with a range of Genz-644131 for 24 h. Cells viability was determined with CellTiter Glo reagent and data were analyzed in GraphPad Prism to determine the IC_50_. Values in parenthesis show the 95% confidence interval. (D) Replicates of western blot analysis in Fig 3C.
(TIF)

S3 Fig Replicates of western blot analysis in Fig 4A. (TIF)

S4 Fig Heat map of total metabolomics replicates of samples from Fig 4 and S3 Fig. Values are on Log_2_ scale.
(TIF)

S5 Fig (A) Steady-state kinetic analysis of recombinant purified *Tb*AdoMetSyn with ATP (left panel) or methionine (right panel) as the variable substrate. Fitted kinetic parameters from these data are showing in Table 1. (B) Replicates of western blot analysis in Fig 5B.
(TIF)

S6 Fig DNA sequence of complement *Tbadometsyn* ORF. Underlined bases indicate the sequence that was altered/scrambled so that the construct would generate mRNA that was resistant to RNAi by the expressed *Tbadometsyn* hairpin sequence.
(TIF)

S7 Fig (A) Growth curve analysis of BSF 427 cells grown in methionine-free HMI-19 with 10% FBS and supplemented with varying levels of methionine. (B) Replicates of western blot analysis in Fig 6B.
(TIF)

S1 Table Prozyme SRM analysis. (XLSX)

S2 Table Metabolomics analysis of 6 h Genz treatment. (XLSX)

S3 Table Methionine measurements from FBS. (XLSX)

S4 Table Primer Table. (XLSX)
