# Supplementary figures and images for: A dual regulatory circuit consisting of S-adenosylmethionine decarboxylase protein and its reaction product controls expression of the paralogous activator prozyme in Trypanosoma brucei
Source: PLoS Pathog. 2018 Oct 26;14(10):e1007404. doi: 10.1371/journal.ppat.1007404 (PMC6221367; doi:10.1371/journal.ppat.1007404)

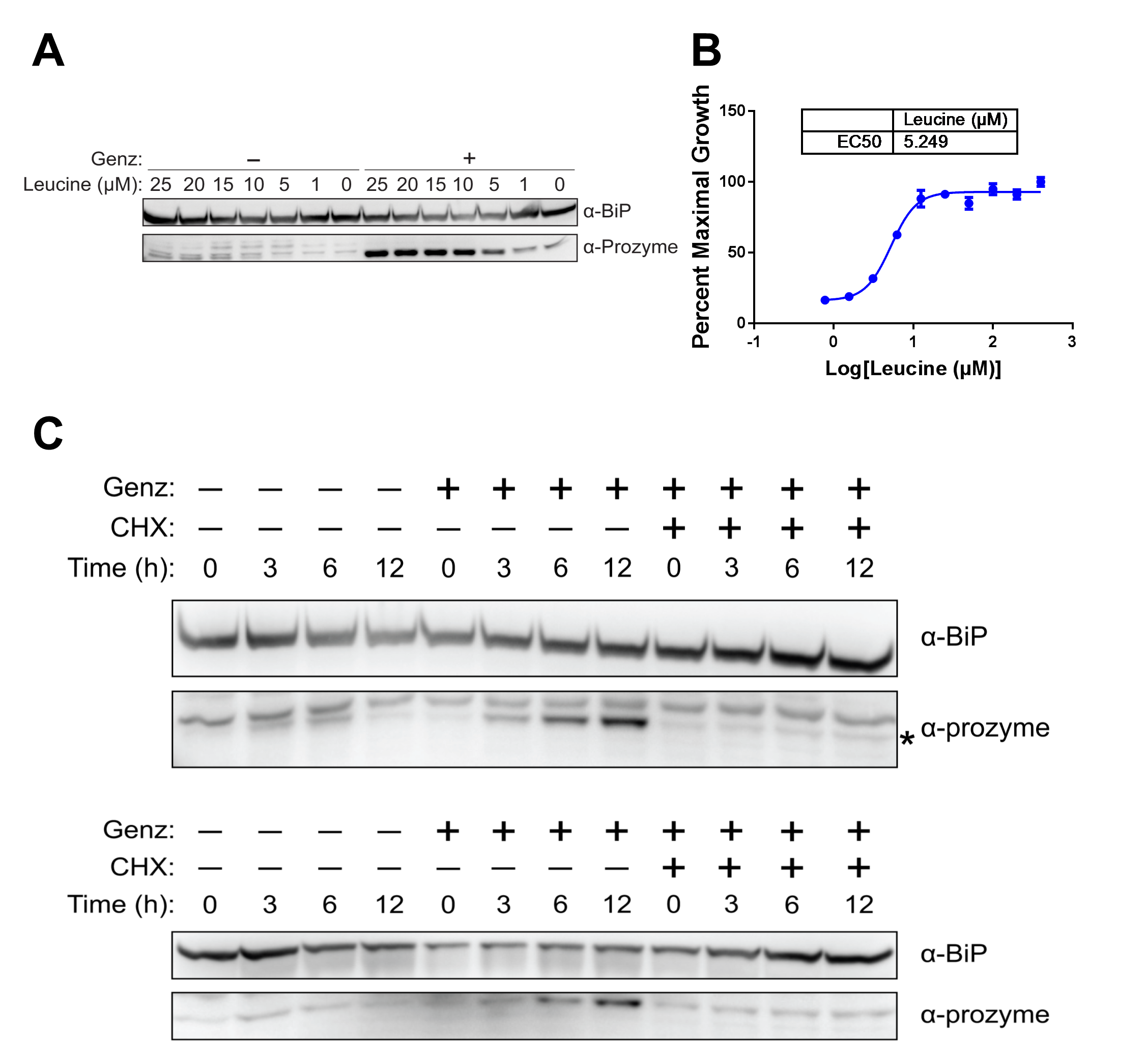

Supplement: S1 Fig — (A) Western blot analysis of BSF 427 cells incubated in leucine-free HMI-19 with 10% dFBS supplemented at varying levels of leucine in the presence or absence of Genz-644131 (15 nM) for 6 h. (B) Cell viability of BSF 427 cells grown for 48 h in leucine-free HMI-19 media with 10% FBS supplemented with varying levels of leucine as indicated. Viable cells were quantitated by CellTiter Glo assay. Data were analyzed in GraphPad Prism to determine the effective concentration at 50% growth (EC50). Error bars represent SD for three biological replicates. (C) Replicates of western blot analysis in Fig 2A. (TIF) [file ppat.1007404.s001.tif]

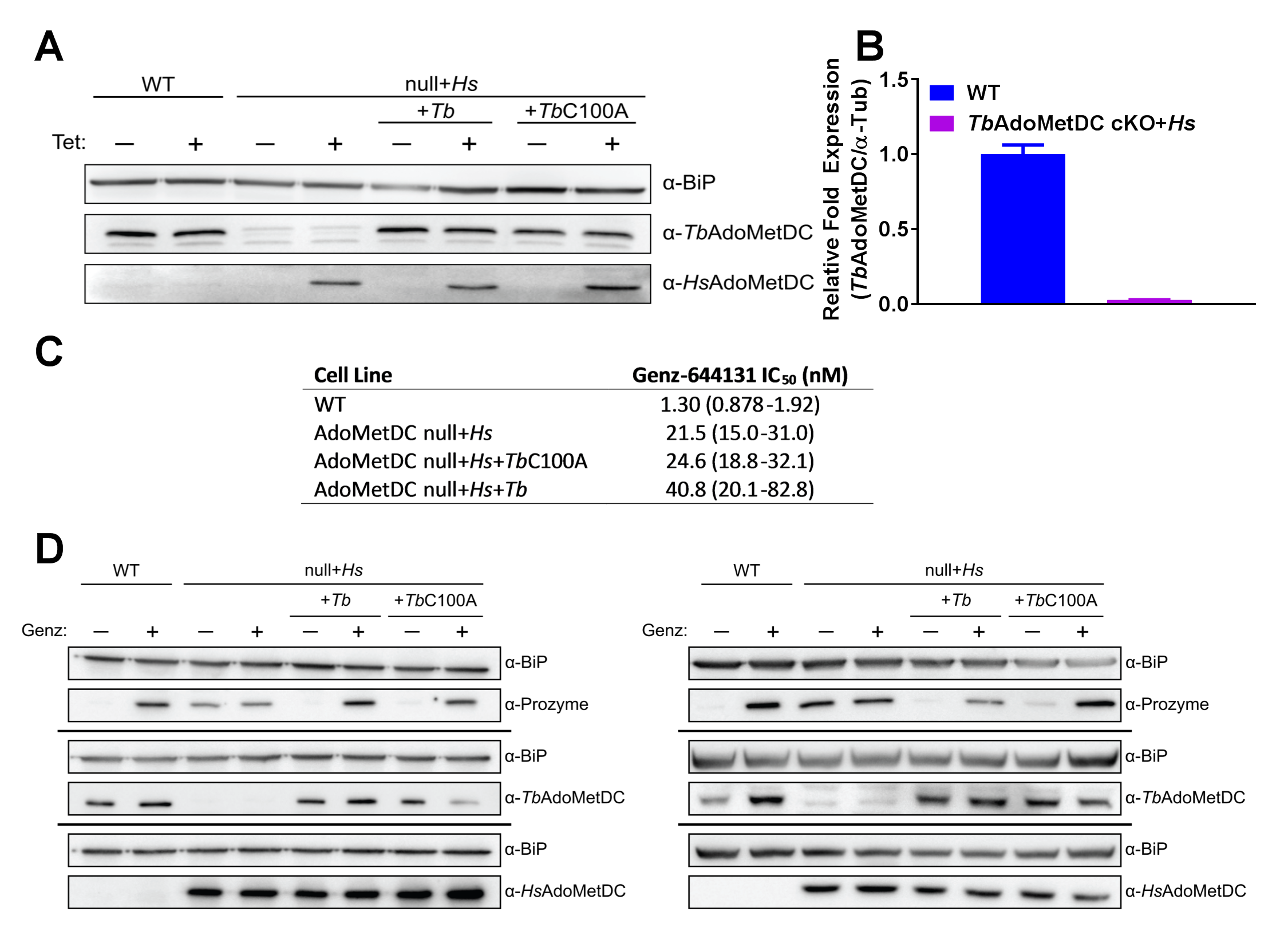

Supplement: S2 Fig — (A) Western blot analysis against TbBiP, TbAdoMetDC, and HsAdoMetDC of WT SM, TbAdoMetDC null+Hs, TbAdoMetDC null+Hs+Tb, TbAdoMetDC null+Hs+TbC100A cell lines cultured ±Tet for 48 h to show TbAdoMetDC and HsAdoMetDC protein levels during growth curve in Fig 3B. (B) RT-qPCR analysis of TbAdoMetDC mRNA from WT SM and TbAdoMetDC null+Hs normalized to α-Tubulin (C) Genz-644131 dose-response analysis of WT SM, TbAdoMetDC null+Hs, TbAdoMetDC null+Hs+Tb, TbAdoMetDC null+Hs+TbC100A cell lines incubated with a range of Genz-644131 for 24 h. Cells viability was determined with CellTiter Glo reagent and data were analyzed in GraphPad Prism to determine the IC50. Values in parenthesis show the 95% confidence interval. (D) Replicates of western blot analysis in Fig 3C. (TIF) [file ppat.1007404.s002.tif]

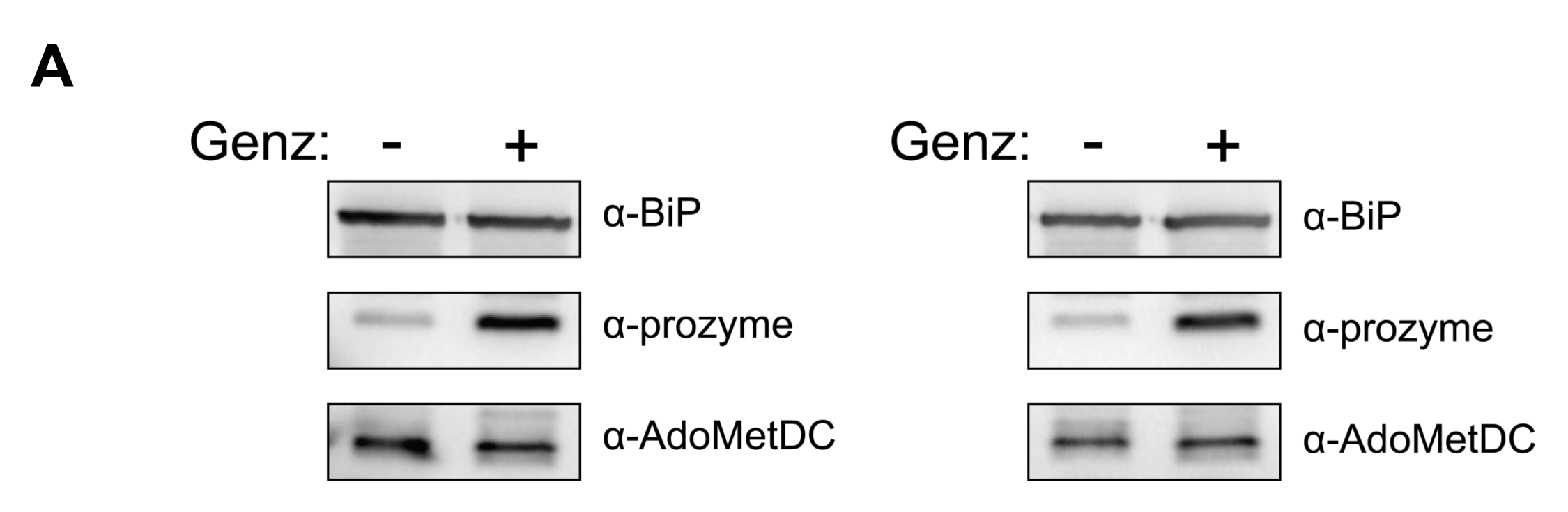

Supplement: S3 Fig — (TIF) [file ppat.1007404.s003.tif]

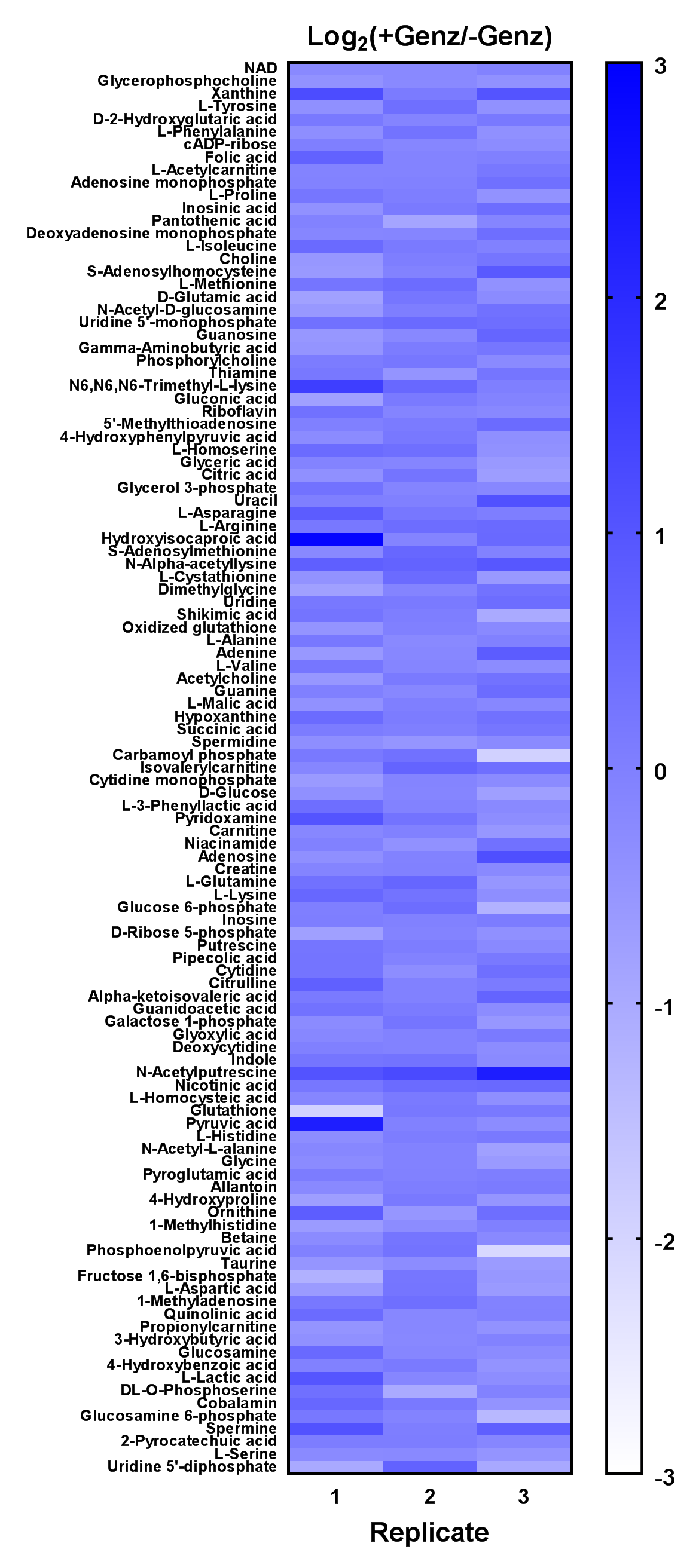

Supplement: S4 Fig — Values are on Log2 scale. (TIF) [file ppat.1007404.s004.tif]

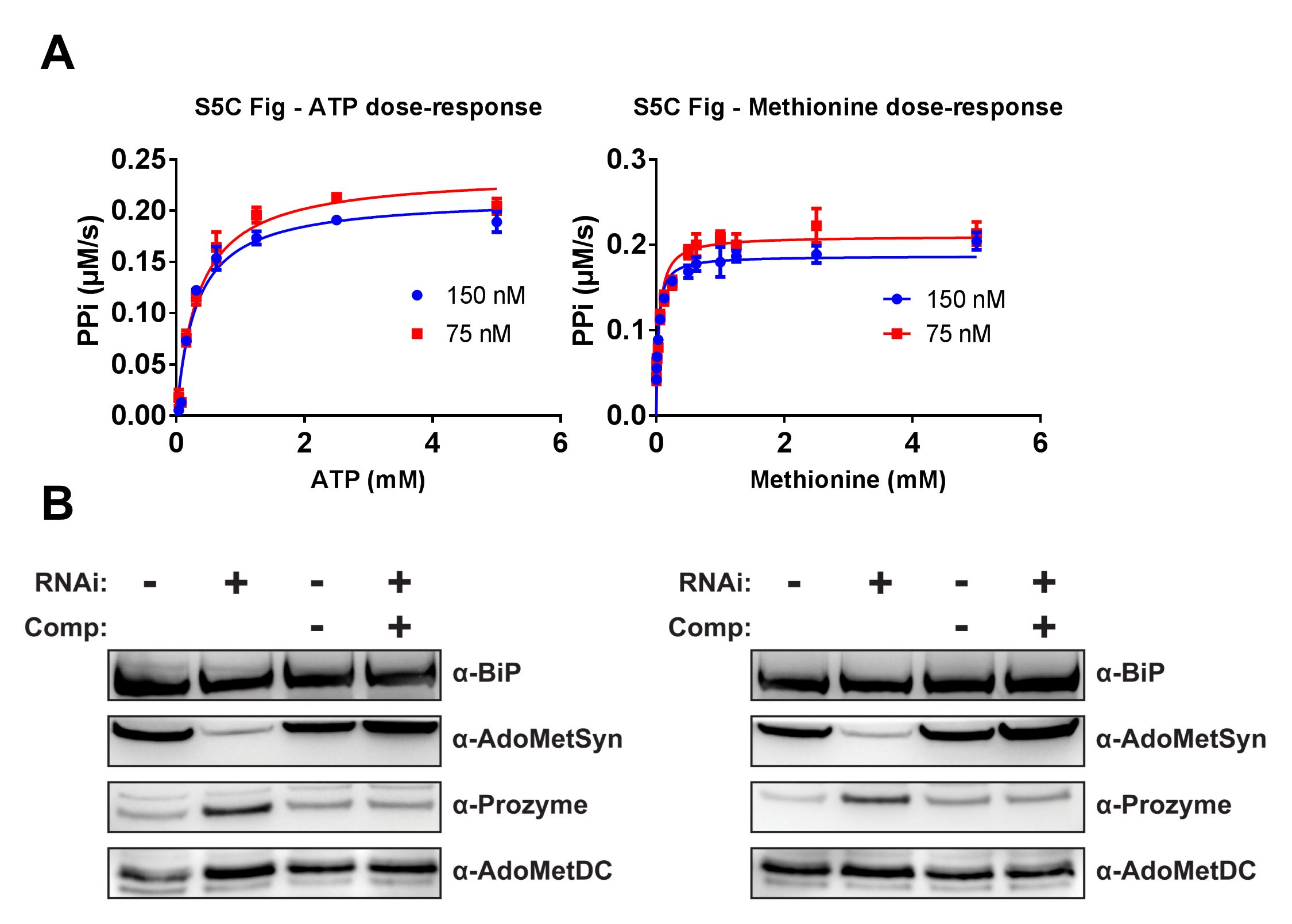

Supplement: S5 Fig — (A) Steady-state kinetic analysis of recombinant purified TbAdoMetSyn with ATP (left panel) or methionine (right panel) as the variable substrate. Fitted kinetic parameters from these data are showing in Table 1. (B) Replicates of western blot analysis in Fig 5B. (TIF) [file ppat.1007404.s005.tif]

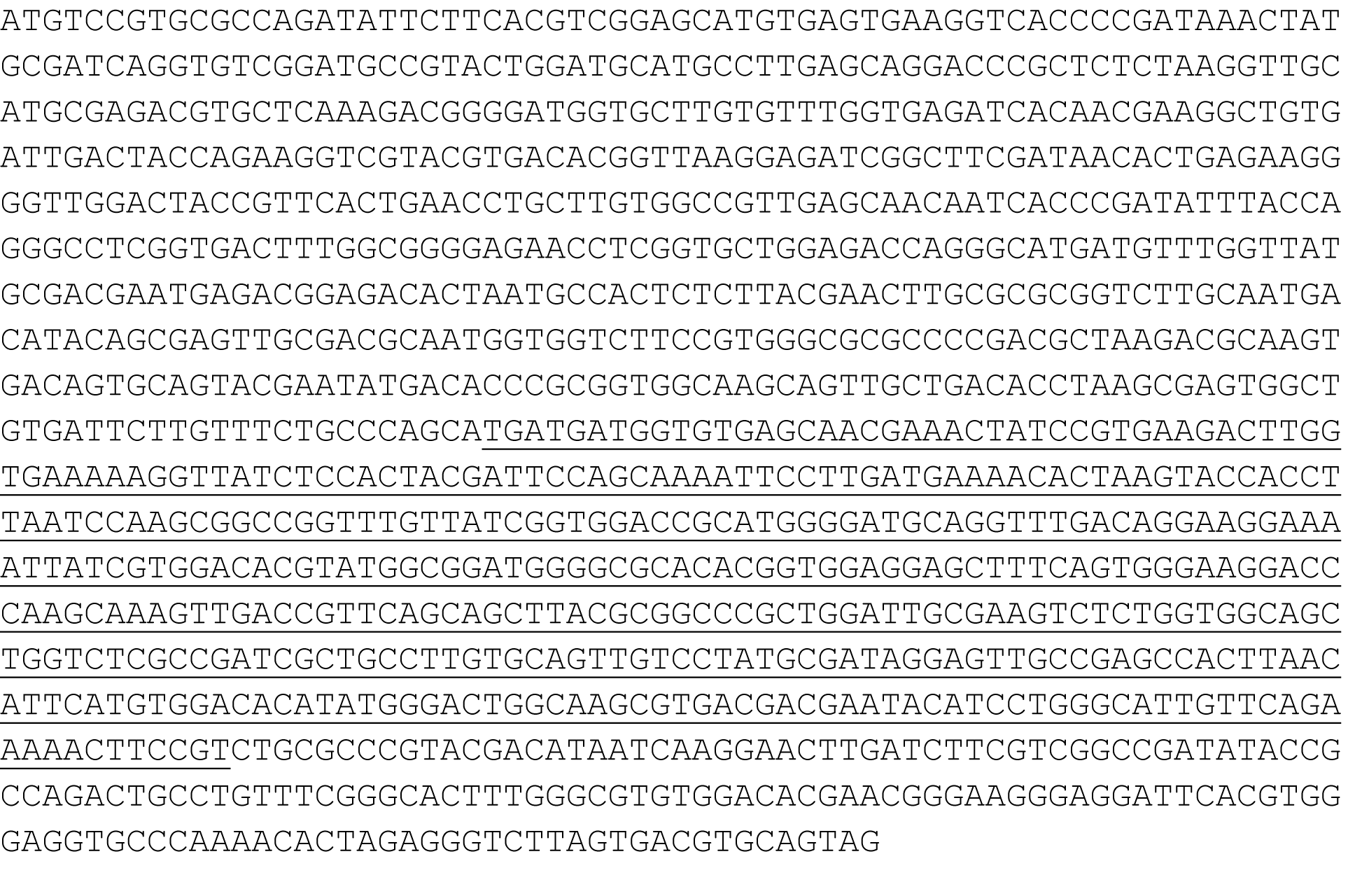

Supplement: S6 Fig — Underlined bases indicate the sequence that was altered/scrambled so that the construct would generate mRNA that was resistant to RNAi by the expressed Tbadometsyn hairpin sequence. (TIF) [file ppat.1007404.s006.tif]

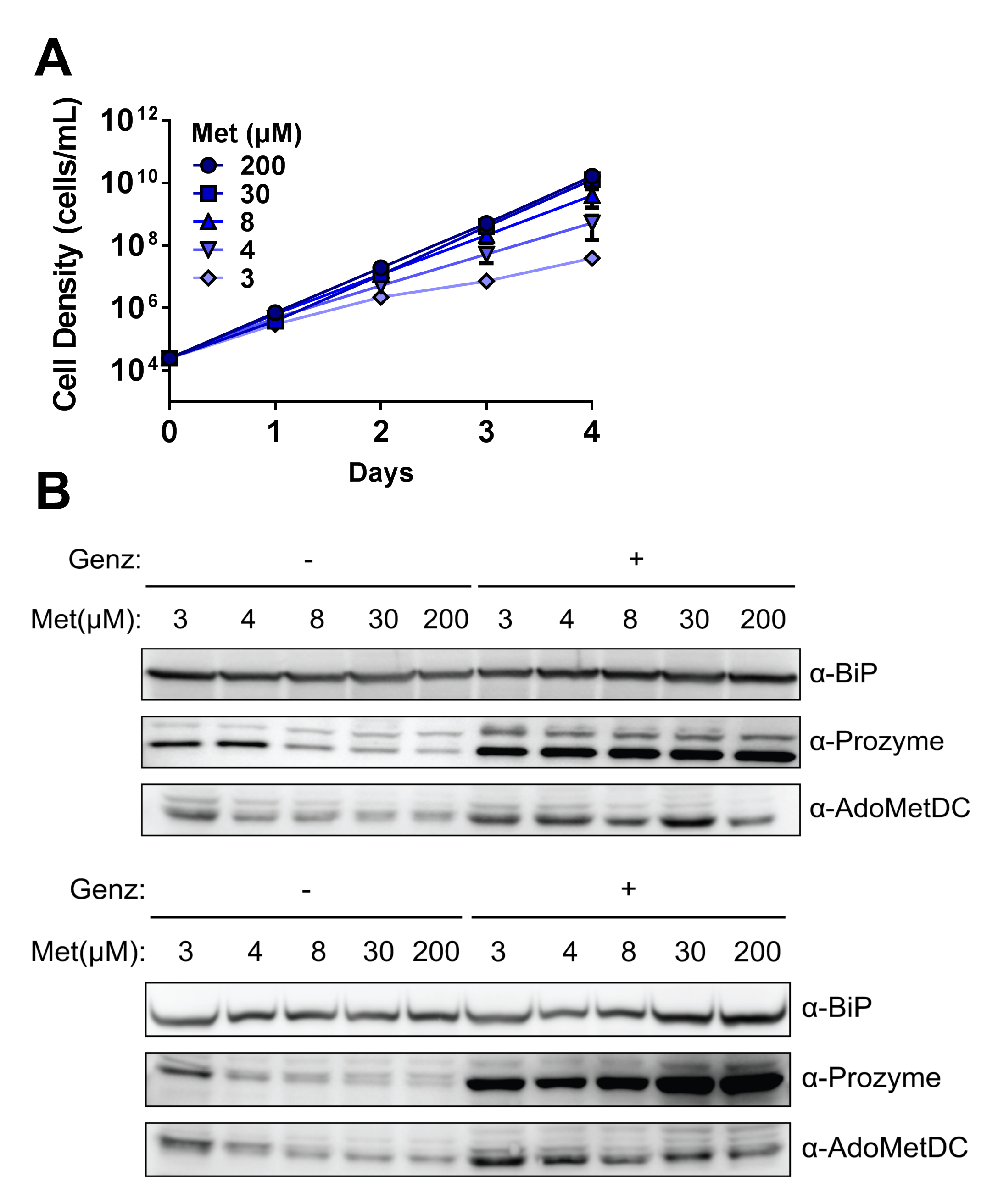

Supplement: S7 Fig — (A) Growth curve analysis of BSF 427 cells grown in methionine-free HMI-19 with 10% FBS and supplemented with varying levels of methionine. (B) Replicates of western blot analysis in Fig 6B. (TIF) [file ppat.1007404.s007.tif]
